# Huang Qin decoction increases SLC6A4 expression and blocks the NFκB-mediated NLRP3/Caspase1/GSDMD pathway to disrupt colitis-associated carcinogenesis

**DOI:** 10.1007/s10142-024-01334-x

**Published:** 2024-03-12

**Authors:** Yili Tao, Lai Wang, Xiaofeng Ye, Xin Qian, Danye Pan, Xiaoyu Dong, Qian Jiang, Po Hu

**Affiliations:** 1 Department of Gastroenterology, Changzhou Hospital of Traditional Chinese Medicine, Changzhou, 213000 Jiangsu P.R. China; 2 Digestive Disease Diagnosis and Treatment Center of Integrated Traditional Chinese and Western Medicine, Changzhou Hospital of Traditional Chinese Medicine, Changzhou, 213000 Jiangsu P.R. China; 3 Department of Pulmonary Diseases, Changzhou Hospital of Traditional Chinese Medicine, Changzhou, 213000 Jiangsu P.R. China

**Keywords:** Pyroptosis, MODE-K cells, Chinese medicine preparation

## Abstract

Huang Qin decoction (HQD) is a traditional Chinese medicine formula for treating colitis, but the effects and molecular mechanism of action of HQD in colitis-associated carcinogenesis (CAC) are still unclear. Therefore, we aimed to determine the beneficial effects of HQD on CAC in mice and to reveal the underlying mechanism involved. AOM/DSS was used to induce CAC in mice, and the effects of HQD on tumorigenesis in mice were examined (with mesalazine serving as a positive control). Mesalazine or HQD treatment alleviated body weight loss and decreased the disease activity index in mice induced by AOM/DSS. Mesalazine or HQD treatment also suppressed the shortening of colon tissue length, the number of tumors, and the infiltration of inflammatory cells. The genes targeted by HQD were predicted and verified, followed by knockout experiments. Elevated SLC6A4 and inhibited serotonin production and inflammation were observed in HQD-treated mice. HQD inhibited the NFκB and NLRP3/caspase1/GSDMD pathways. The therapeutic effect of HQD was diminished in SLC6A4-deficient AOM/DSS mice. Additionally, the downregulation of SLC6A4 mitigated the inhibitory effect of HQD-containing serum on MODE-K cell pyroptosis. Our findings suggest that SLC6A4 is a pivotal regulator of HQD-alleviated CAC via its modulation of the NLRP3/caspase1/GSDMD pathway.

**Supplementary Information:**

The online version contains supplementary material available at 10.1007/s10142-024-01334-x.

## Introduction

Patients with chronic inflammatory bowel diseases are at an increased risk of developing colitis-associated carcinogenesis (CAC), and the cumulative incidence of developing CAC increases with prolonged colon inflammation (Hirano et al. 2020; Saraggi et al. 2017). The occurrence and development of ulcerative colitis are closely related to intestinal inflammatory responses and immune responses, and treating ulcerative colitis with Western medicine is often associated with adverse outcomes (Zheng et al. 2022). Treatments for ulcerative colitis include 5-aminosalicylic acid (5-ASA), steroids, and immunosuppressants (Ungaro et al. 2017). Mesalazine, a 5-ASA agent, has a well-established role in the management of active and inactive mild-to-moderate ulcerative colitis (Criscuoli et al. 2013). Even though 5-ASA has a lower rate and greater severity of side effects than other therapies, there is evidence suggesting its low effectiveness (Veloso et al. 2021).

There are positive signs that traditional Chinese medicine (TCM) can regulate inflammatory cytokines and the immune system, thereby helping to treat ulcerative colitis (Liu et al. 2022). Moreover,, the relevance of Huang Qin decoction (HQD) for the suppression of CAC has recently been highlighted (Pan et al. 2022; Zhou et al. 2022). However, many questions remain unanswered regarding the underlying mechanism involved. HQD consists of four herbs: *Scutellaria baicalensis*, *Paeonia lactiflora*, *Glycyrrhiza uralensis*, and *Ziziphus jujuba* (Wu et al. 2022). Integrated bioinformatics prediction revealed that androgen receptor (AR), estrogen receptor (ESR1), and solute carrier family 6 member 4 (SLC6A4, also called serotonin reuptake transporter, [SERT]) might be three candidates targeted by HQD and these four components. Interestingly, human SLC6A4, which maps to chromosome 17q11.2, exerts many gastrointestinal effects under both normal and pathological conditions (Gonzalez Delgado et al. 2022; Murphy and Moya 2011). Serotonin has been reported to activate nuclear factor kappa B (NFκB) signaling in concanavalin A-mediated liver injury (Pang et al. 2020). Furthermore, the gasdermin (GSDM) family of proteins, which are downstream effectors of the inflammasome that are known primarily for their function in pyroptosis, has been recently linked to the pathogenesis of colorectal cancer (Privitera et al. 2021). NFκB is a critical transcription factor that upregulates NLRP3 synthesis, and NLRP3 interacts with the adaptor ASC, which recruits the effector pro-caspase-1, leading to the formation of the NLRP3 inflammasome, within which pro-caspase-1 undergoes self-cleavage and becomes capable of processing the interleukin (IL) precursors pro-IL-1β and pro-IL-18 into their mature forms (Afonina et al. 2017). Therefore, we hypothesized that the inhibitory effects of HQD on CAC are partially mediated by the regulation of SLC6A4 and the subsequent NFκB-mediated NLRP3/Caspase1/GSDMD pathway. This hypothesis was tested by using an azoxymethane/dextran sodium sulfate (AOM/DSS)-induced mouse model and a murine intestinal epithelial cell line MODE-K treated with HQD or the drug-contained serum (DS).

## Materials and methods

### Composition and preparation of HQD

Dried Chinese herbal medicines were selected according to the 2020 edition of the Chinese Pharmacopoeia and purchased by the Pharmacy Department of Changzhou Hospital of Traditional Chinese Medicine in March 2022 from Bozhou Traditional Chinese and Western Medicine Pharmaceutical Co. (Bozhou, Anhui, China). The herbs were identified by the Pharmacy Department and all voucher specimens (CF-202203-5) were deposited at Changzhou Hospital of Traditional Chinese Medicine. *Scutellaria baicalensis* (9 g), *Paeonia lactiflora* (6 g), *Glycyrrhiza uralensis* (6 g), and *Ziziphus jujuba* (49 g) were soaked in 10 volumes the volume of distilled water for 30 min, and boiled at 100 °C for 30 min. The liquid was filtered, and the residue was extracted using 8 times the volume of water. The two filtrates were combined and concentrated into a 1 g/mL herbal decoction after filtration through a membrane and stored at 4 °C for subsequent analysis (Mo et al. 2022).

### Network pharmacology

BATMAN-TCM (http://bionet.ncpsb.org.cn/batman-tcm/index.php/Home/Index/index): an enhanced integrative database for known and predicted linkages between TCM ingredients and target proteins (Kong et al. 2024), was used to analyze the ingredient-target-pathway/disease network for HQD. The possible target genes of its main components (*Scutellaria baicalensis*, *Paeonia lactiflora*, *Glycyrrhiza uralensis*, and *Ziziphus jujuba*) were subsequently analyzed using the Chinese herbal medicine database HERB 2.0: A high-throughput experiment- and reference-guided database of TCM (http://herb.ac.cn/). The targets common to both BATMAN-TCM and HERB 2.0 were obtained through the Jvenn website (https://jvenn.toulouse.inrae.fr/app/example.html).

### Animals and experimental design

C57BL/6J wild-type (WT) mice were purchased from Beijing Vital River Laboratory Animal Technology Co., Ltd. (Beijing, China), and SLC6A4-knockout (SLC6A4-KO) mice with a C57BL/6J genetic background were purchased from Cyagen Biosciences (Guangzhou, Guangdong, China). Mice were maintained under specific pathogen-free (SPF) conditions (The SPF environment complies with the national standard of the People’s Republic of China GB14922-2022: *Laboratory animal-microbiological and parasitical standards and monitoring* and all mice do not carry pathogens that pose a significant health risk to animals and/or interfere with scientific research) for one week before the experiment. All the procedures were in strict accordance with the P.R. China legislation on the use and care of laboratory animals. The experimental protocols were approved by the Animal Ethics Committee of Changzhou Hospital of Traditional Chinese Medicine (Approval No.: 2022-017).

C57BL/6J mice were divided into 6 groups (*n* = 6): the control, AOM/DSS, AOM/DSS + Mesalazine, AOM/DSS + low (L)-HQD (2.275 g/kg), AOM/DSS + medium (M)-HQD (4.55 g/kg), AOM/DSS + high (H)-HQD (9.1 g/kg) groups. Except for those in the control group, the mice in the other groups were injected intraperitoneally with 10 mg/kg of AOM (one time, A885948, Shanghai Macklin Biochemical Co., Ltd., Shanghai, China). One week later, 2.5% DSS (D806297, Macklin) was added to the drinking water for one week. This was followed by a return to normal drinking water treatment for two weeks. One cycle of treatment was administrated for three weeks, and the cycle was repeated three times. The body weights of the mice were measured weekly, and the disease activity index (DAI) was evaluated at the beginning of the first cycle of DSS. Euthanasia by intraperitoneal injection of sodium pentobarbital (Sinopharm Chemical Reagent Co., Ltd., Shanghai, China) was conducted at the end of the third cycle or when the mice developed anal prolapse and/or lost ≥ 20% of their body weight. For the control group, the mice were injected intraperitoneally or gavaged with the corresponding volume of saline and normal drinking water, respectively. For treatment with mesalazine and HQD, mesalazine (200 mg/kg; National Drug Code H19980148; Lulingpharm, Jiamusi, Heilongjiang) (Liang et al. 2019) and HQD (2.275, 4.55, and 9.1 g/kg) (Li et al. 2019) were administered by gavage daily after one week of AOM treatment. The dose of mesalazine was converted from a mouse dose to a human equivalent dose by the body surface area method as = (200 mg/kg × 20 g)/(70 kg × 0.0026) = 4 mg/0.182 kg = 22.0 mg/kg, which follows the recommended daily dose of UC in humans (70 kg) established by the Chinese Pharmacopoeia Commission (1.5 – 4 g = 21.4 mg/kg − 57.1 mg/kg).

For comparison of WT mice and SLC6A4-KO mice (*n* = 6/group), both groups of mice were induced with AOM/DSS. Considering that a high concentration of HQD had the greatest therapeutic effect on AOM/DSS mice, a high concentration of HQD (9.1 g/kg) was used for the following assays.

### Evaluation of colitis

The DAI was used to assess the severity of colitis. The DAI was assessed by a combination of weight loss (percentage), stool consistency, and blood in the stool. The scoring criteria were as follows: (1) body weight loss (0: < 1%, 1: 1–5%, 2: 5–10%, 3: 10–15%, 4: > 15%); stool consistency (0: normal, 2: loose stool, 4: diarrhea); blood in the stool (0: no bleeding, 2: slight bleeding, 4: gross bleeding) (Tajasuwan et al. 2022).

### Histological staining

Mouse colon tissues were fixed overnight in Bouin’s fixative, embedded in paraffin, and sectioned. Paraffin-embedded sections were dewaxed with xylene, hydrated with gradient alcohol, and stained with hematoxylin-eosin (HE) staining solution (E607318, Shanghai Sangon Biological Engineering Technology & Services Co., Ltd., Shanghai, China). After the staining was completed, the sections were dehydrated and sealed with neutral resin after being cleared in xylene. Finally, the pathological morphology of mouse colon tissues was observed under a microscope (Wang et al. 2023).

### Enzyme-linked immunosorbent assay (ELISA)

The levels of 5-hydroxytryptamine (5-HT), IL-1β, and IL-18 in mouse colon tissues were measured with 5-HT (AD3266Mo, Beijing Andy Huatai Technology Co., Ltd., Beijing, China), IL-1β (JYM0531Mo, Jiyinmei, Wuhan, Hubei, China), and IL-18 (JYM0543Mo, Jiyinmei) kits. Briefly, the optical density (OD) value at 450 nm in each well was measured according to the instructions, and the corresponding concentration in each well was calculated from the standard curve.

### RT-quantitative polymerase chain reaction (RT-qPCR) analysis

TRIzol (15,596,026, Invitrogen Inc., Carlsbad, CA, USA) was used for the extraction of mRNA from mouse colon tissues and cells. RNA was subsequently reverse transcribed using a cDNA synthesis kit (11141ES10; Yeasen Biotechnology Co., Ltd., Shanghai, China). The mRNA expression was subsequently detected using Hieff UNICON Universal Blue qPCR SYBR Green Master Mix (11184ES03, Yeasen). The data shown are the relative abundances of SLC6A4 normalized to the abundance of β-actin. qPCR was performed using the following primers: SLC6A4: 5’-GTTGATGCTGCGGCTCAGATCT-3′ (forward) and 5′-GAAGCTCGTCATGCAGTTCACC-3′ (reverse); β-actin: 5′-CATTGCTGACAGGATGCAGAAGG-3′ (forward) and 5′-TGCTGGAAGGTGGACAGTGAGG-3′ (reverse).

### Western blot

RIPA lysis buffer (89,901; Thermo Fisher Scientific Inc., Waltham, MA, USA) was used for the extraction of total protein from tissue and cells. Total protein was quantified using a BCA quantification kit (23,227, Thermo Fisher). The proteins were separated by sodium dodecyl sulfate-polyacrylamide gel electrophoresis and transferred to polyvinylidene fluoride (PVDF) membranes by the wet transfer method. After being blocked with skim milk for 45 min at room temperature, the PVDF membranes were incubated overnight at 4 °C with primary antibodies against SLC6A4 (1:1000, H00006532-D01P, Novus Biological Inc., Littleton, CO, USA), p-NFκB p65 (1:1000, #3033, Cell Signaling Technologies, Beverly, MA, USA), NFκB p65 (1:1000, #8242, Cell Signaling Technologies), NLRP3 (1:1000, #15,101, Cell Signaling Technologies), Cleaved-Caspase-1 P20 (1:1000, PA5-99390, Invitrogen), Cleaved-GSDMD (1:1000, #50,928, Cell Signaling Technologies), GSDMD (1:1000, ab225867, Abcam, Cambridge, UK), Caspase1 (1:1000, ab138483, Abcam), ASC (1:1000, #67,824, Cell Signaling Technologies), and β-actin (1:200, ab115777, Abcam). The following day, the PVDF membranes were washed and incubated with an HRP-conjugated secondary antibody (1:5000, ab205718, Abcam) for 1 h at room temperature. Finally, immunoreactive protein bands were detected by the high sensitive ECL luminescence reagent (C500044, Sangon), and β-actin was used as an internal reference to analyze the relative protein expression.

### Cell culture and lentivirus infection

Murine intestinal epithelial MODE-K cells were purchased from BLUEFBIO (Shanghai, China) and maintained in Dulbecco’s modified Eagle’s medium supplemented with 10% fetal bovine serum and 1% penicillin and streptomycin. The cells were incubated in a 5% CO_2_ incubator at 37 °C. The sh-NC and sh-SLC6A4 lentiviruses used for infection were purchased from Obio (Shanghai, China). Lentivirus infection was conducted by seeding the cells into culture plates and adding the lentivirus solution when the cell density reached 60%. Antibiotics were added 48 h after infection to screen for lentivirus-infected cells.

### DS preparation

Sprague Dawley rats (from the Vital River) were divided into the control and DS groups via gavage of saline or HQD (9.1 g/kg) for 7 consecutive days. Blood was collected from the rats 3 h after the last gavage. The sera were separated by centrifugation at 3000 rpm for 15 min and inactivated at 56 °C for 30 min, followed by filtration through 0.22-µm filter membranes. The collected serum was stored at -20 °C for cell treatment (Zhu et al. 2019).

### Development of an in vitro pyroptosis model and treatment with DS

MODE-K cells were treated with 100 ng/mL LPS (AC11974, Shanghai Acmec Biochemical Co., Ltd., Shanghai, China) for 4 h and then stimulated with 5 mM ATP (A832633, Macklin) for 30 min to induce pyroptosis (Chen et al. 2019). For pretreatment with DS, MODE-K cells were cultured in DMEM supplemented with 10% DS for 24 h before the LPS/ATP induction. The control cells were cultured with control serum.

### Cell viability assay

MODE-K cells were assayed for cell viability with a cell counting kit-8 (CCK-8, HY-K0301, MedChemExpress, Monmouth Junction, NJ, USA). Briefly, 10 µL of CCK-8 solution was added to each well, and the cells were incubated for 2 h in an incubator according to the manufacturer’s instructions. Finally, the optical density (OD) values at 450 nm were measured by a microplate reader.

### Lactate dehydrogenase (LDH) release assay

LDH kits (C0016, Beyotime Biotechnology Co., Ltd., Shanghai, China) were used to measure the release of LDH from cells. The sample maximum enzyme activity control wells were added to the LDH release reagent provided in the kit as a positive control. The cells were centrifuged at 500 ×*g* for 6 min for precipitation, after which the cell supernatant of cells was collected. The configured LDH assay solution was added for a 20-min incubation in the dark, and the OD_490_ values of each group were measured using a microplate reader. The release of LDH was calculated according to the following formula: (OD value of treated samples - OD value of sample control wells) / (OD value of positive control wells – OD value of sample control wells) × 100.

### Immunofluorescence

The cells were fixed with paraformaldehyde, permeabilized in Triton X-100 for 20 min, blocked with goat serum for 45 min, and incubated with a diluted primary antibody against ASC (1:500, #67,824; Cell Signaling Technologies) overnight at 4 °C. After the cells were incubated with an Alexa Fluor 488-conjugated secondary antibody (1:1000; #4412; Cell Signaling Technologies) for 2 h in the dark, the cell slides were stained with DAPI, mounted, and then imaged under a fluorescence microscope. The fluorescence intensity of ASC in the cells was observed (Qu et al. 2022).

### Statistics

Statistical analysis in this study was performed with Prism 8.0 (GraphPad, San Diego, CA, USA), and the data are presented as mean ± SDs. The unpaired t-test was used to compare the two groups. For multigroup comparisons, the data were analyzed by one-way or two-way ANOVA and Tukey’s multiple comparison tests. Each experiment was conducted with biological and technical replicates with three replications of the entire experiment unless otherwise specified. Differences were considered to be significant at a p-value of less than 0.05.

## Results

### HQD inhibits CAC

**Fig. 1:**
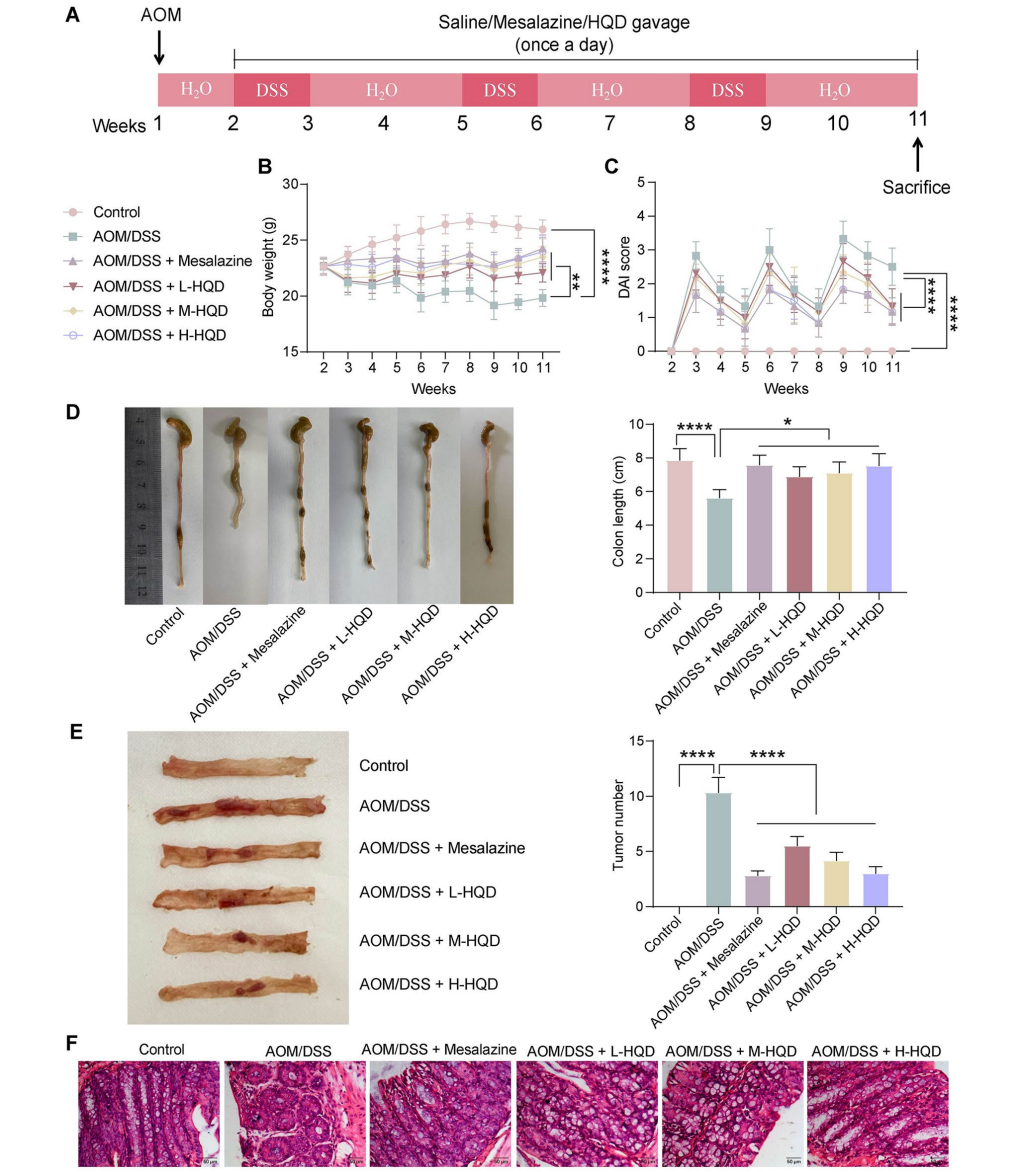
HQD ameliorates CAC in mice induced by AOM/DSS. (A) Scheme of AOM/DSS-induced CAC and treatment with mesalazine (positive control), L-HQD (2.275 g/kg), M-HQD (4.55 g/kg), and H-HQD (9.1 g/kg). (B) Changes in body weight of mice. (C) DAI of the mice. (D) Length of colon tissue in mice. (E) Number of tumors in the colon tissues of the mice. (F) HE staining of the pathological morphology of the colon tissues. The data are presented as the means ± SDs (*n* = 6). *p < 0.05, **p < 0.01, ****p < 0.0001. The data were analyzed by one-way or two-way ANOVA and Tukey’s multiple comparison test

AOM/DSS model mice were developed and treated with mesalazine or low, medium, and high concentrations of HQD (named L-HQD, M-HQD, and H-HQD, respectively) (Fig. 1A). At the later stage of the experiment, AOM/DSS mice lost weight, while mesalazine or HQD treatment alleviated the weight loss of AOM/DSS mice. Notably, the effects of mesalazine and H-HQD were greater (Fig. 1B). DAI was elevated in AOM/DSS mice compared with controls, and mesalazine or HQD treatment reduced DAI in the colon tissues of AOM/DSS mice (Fig. 1C). Mesalazine or HQD treatment attenuated the shortening of colon tissue in AOM/DSS mice (Fig. 1D). In addition, mesalazine or HQD treatment reduced the number of tumors in AOM/DSS mice (Fig. 1E). HE staining was performed to evaluate the morphology of the colon tissues from each group of mice. Compared with those in the control group, the recesses in the colon tissues of AOM/DSS mice were damaged, with irregular distribution of glands and infiltration of inflammatory cells. In contrast, mesalazine or HQD treatment improved the glandular structure and reduced inflammatory cell infiltration (Fig. 1F).

### HQD promotes SLC6A4 expression and reduces the serotonin concentration in AOM/DSS-induced mice

**Fig. 2:**
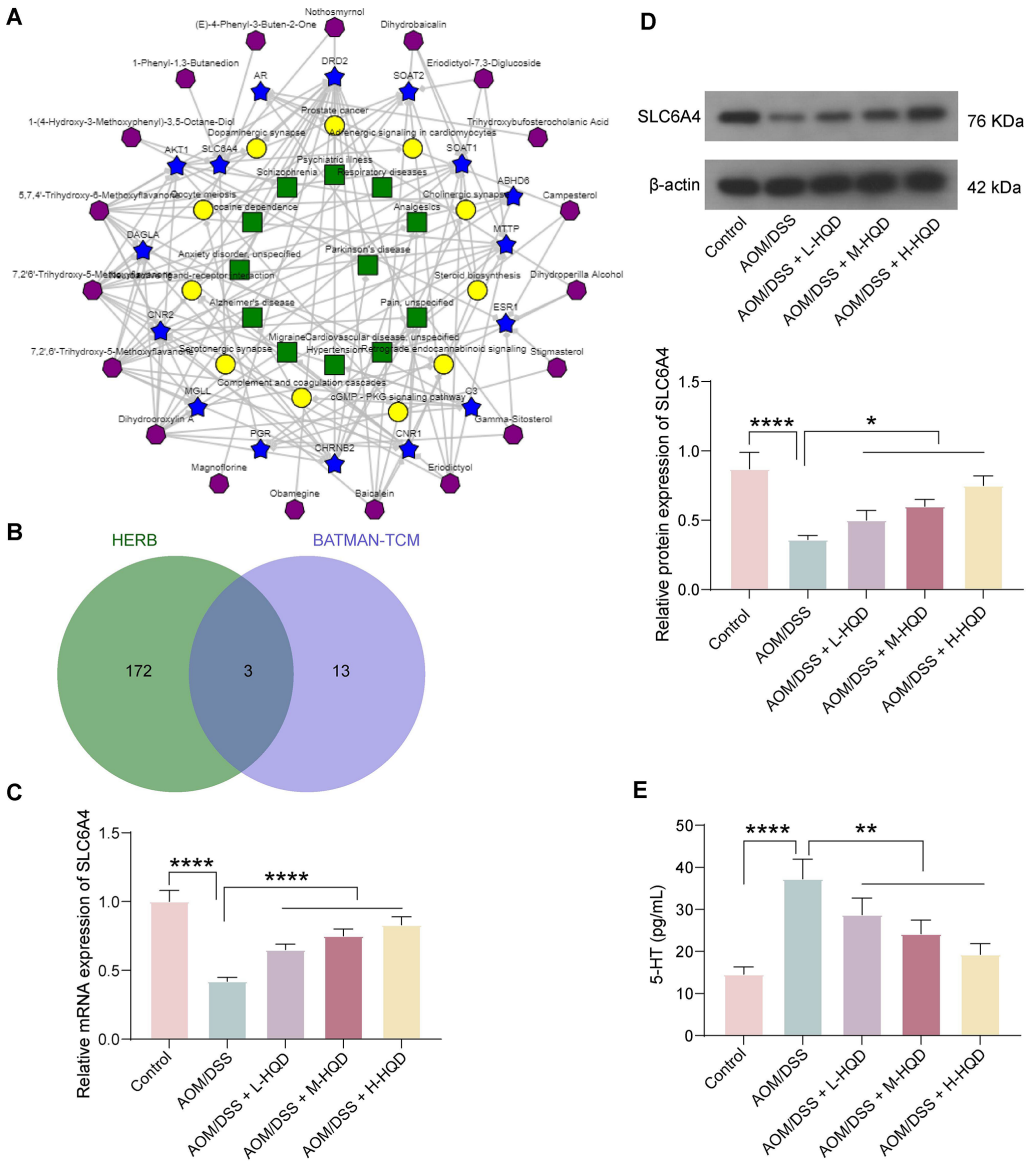
HQD affects SLC6A4 expression and serotonin production in mouse colon tissues. (A) The ingredient-target-pathway/disease network of HQD in BATMAN-TCM. (B) The intersection of therapeutic targets screened in BATMAN-TCM and genes affected by *Scutellaria baicalensis*, *Paeonia lactiflora*, *Glycyrrhiza uralensis*, and *Ziziphus jujuba* in HERB 2.0. (C) Changes in SLC6A4 mRNA expression in AOM/DSS mice after HQD treatment detected by RT-qPCR. (D) Changes in SLC6A4 protein expression in AOM/DSS mice after HQD treatment detected by western blot assays. (E) Detection of serotonin (5-HT) production in mouse colon tissues by ELISA. The data are presented as the means ± SDs (*n* = 6). *p < 0.05, **p < 0.01, ****p < 0.0001. The data were analyzed by one-way ANOVA and Tukey’s multiple comparison test

The intersection of therapeutic targets of HQD in different diseases (Fig. 2A) and the gene expression affected by the main components of HQD yielded 3 intersecting targets: AR, ESR1, and SLC6A4 (Fig. 2B). The serotonin transporter coded by the SLC6A4 gene has been implicated in the pathogenesis of inflammatory bowel disease which includes ulcerative colitis (Gonzalez Delgado et al. 2022). Therefore, we hypothesized that SLC6A4 inhibits CAC by terminating the action of serotonin. SLC6A4 expression was determined in the colon tissues of AOM/DSS mice after HQD treatment using RT-qPCR and western blot assays. The expression of SLC6A4 was decreased in the colon tissues of AOM/DSS mice and increased after HQD treatment (Fig. 2C, D). Serotonin (5-HT) is considered to be involved in CAC (Mao et al. 2022). HQD decreased the serotonin concentration in the colon tissues of AOM/DSS mice compared to AOM/DSS mice without treatment (Fig. 2E).

### HQD inhibits NFκB pathway-mediated cell pyroptosis and inflammation

**Fig. 3:**
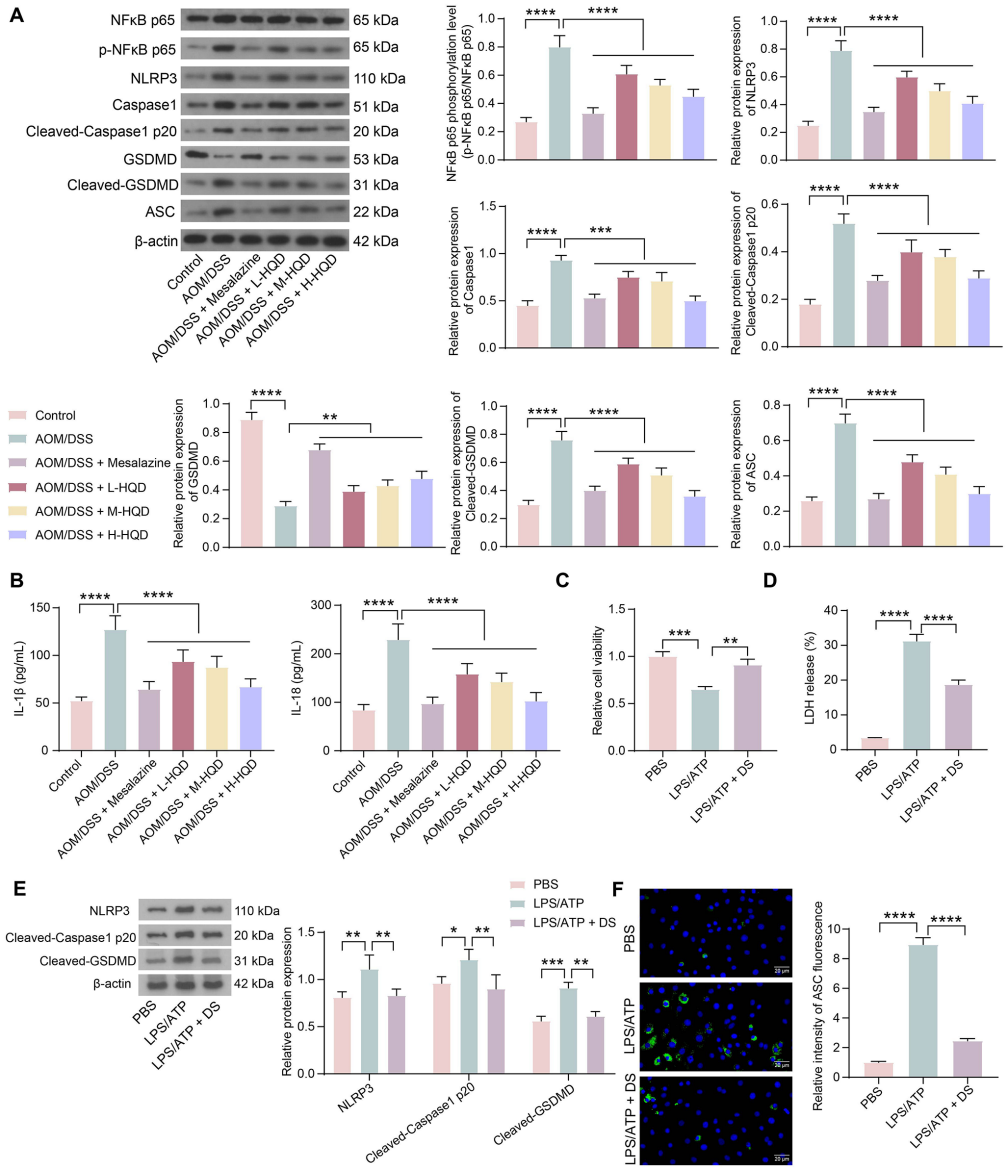
HQD blocks the NLRP3/Caspase1/GSDMD pyroptosis pathway by impairing the NFκB pathway. (A) The expression of the p-NFκB p65, NLRP3, Cleaved-Caspase1 P20, Cleaved-GSDMD, and ASC proteins in the colon tissue of AOM/DSS mice was determined by western blot assay. (B) Detection of IL-1β and IL-18 levels in mouse colon tissues by ELISA. MODE-K cells were pretreated with DS and induced with LPS/ATP. (C) MODE-K cell viability was examined using a CCK-8 assay. (D) Measurement of LDH release. (E) The expression of NLRP3, Cleaved-Caspase1 P20, and Cleaved-GSDMD in MODE-K cells was examined using a western blot analysis. (F) Immunofluorescence detection of ASC expression in MODE-K cells. The data are presented as the means ± SDs (*n* = 6 or 3). *p < 0.05, **p < 0.01, ***p < 0.001, ****p < 0.0001. The data were analyzed by one-way or two-way ANOVA and Tukey’s multiple comparison test

To investigate whether HQD inhibits the NLRP3/Caspase1/GSDMD pyroptosis pathway through inhibition of the NFκB pathway, we examined the expression of relevant proteins by western blot assays (Fig. 3A). Significantly increased NFκB p65 phosphorylation in AOM/DSS mice resulted in increased expression of NLRP3 inflammasome-associated proteins (NLRP3, ASC, and Caspase1), which mediated Cleaved-Caspase1 P20 and subsequent Cleaved-GSDMD. Mesalazine or HQD treatment inhibited the phosphorylation of NFκB p65, suppressed inflammasome activation, and reduced the expression of Cleaved-Caspase1 P20 and Cleaved-GSDMD in the colon tissues of AOM/DSS mice. ELISA was also conducted to measure the inflammatory cytokines IL-1β and IL-18 in mouse colon tissues. Mesalazine or HQD treatment reduced the production of IL-1β and IL-18 in mouse colon tissues (Fig. 3B).

To investigate the effect of HQD on cell pyroptosis in vitro, we established a cell pyroptosis model in MODE-K cells by LPS and ATP stimulation and examined the effect of DS pretreatment. The CCK-8 assay showed an increase in MODE-K cell viability in the DS pretreatment group (Fig. 3C). In addition, LDH levels induced by LPS/ATP treatment were partially decreased in the supernatant of DS-pretreated cells (Fig. 3D). Western blot analysis of NLRP3, Cleaved-Caspase1 P20, and Cleaved-GSDMD protein expression in cells revealed that DS inhibited the expression of pyroptosis-related proteins induced by LPS/ATP (Fig. 3E). Immunofluorescence was carried out to examine the expression of ASC. The fluorescence intensity of ASC was increased in MODE-K cells stimulated with LPS/ATP, and DS pretreatment suppressed the increase in ASC fluorescence intensity (Fig. 3F).

### The inhibitory effect of HQD on CAC is diminished in SLC6A4-deficient mice

**Fig. 4:**
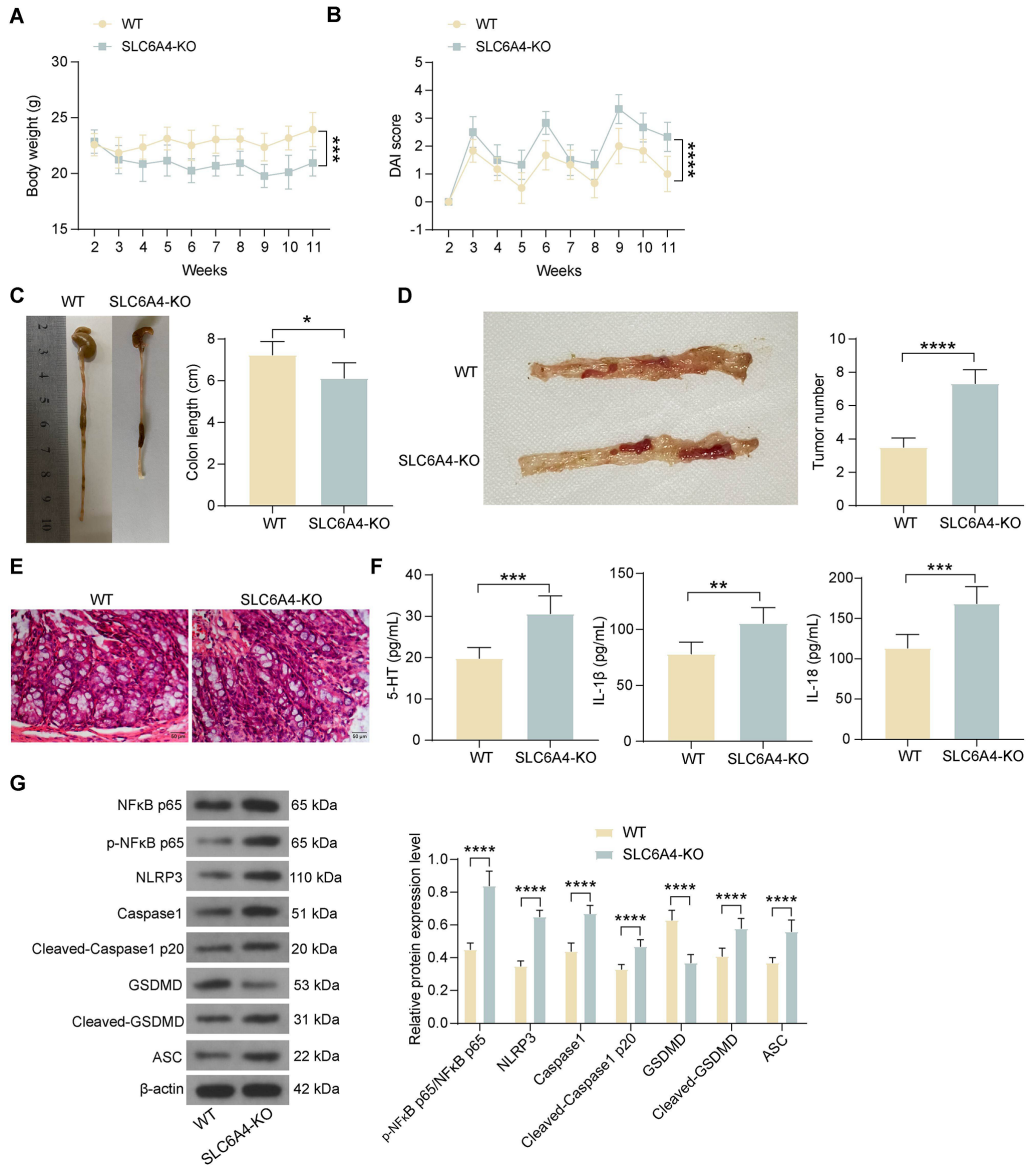
The therapeutic effect of HQD on AOM/DSS mice is inhibited after SLC6A4 knockout. AOM/DSS was used to establish WT and SLC6A4-deficient mice, which were subsequently treated with HQD. (A) Changes in the body weight of mice. (B) DAI of the mice. (C) Length of colon tissue in mice. (D) Number of tumors in the colon tissues of the mice. (E) HE staining of pathological morphology of the colon tissues. (F) Detection of 5-HT, IL-1β and IL-18 levels in mouse colon tissues by ELISA. (G) The expression of the p-NFκB p65, NLRP3, Cleaved-Caspase1 P20, Cleaved-GSDMD, and ASC proteins was determined in the colon tissue of AOM/DSS mice by western blot assay. The data are presented as the means ± SDs (*n* = 6). *p < 0.05, **p < 0.01, ***p < 0.001, ****p < 0.0001. The data were analyzed by an unpaired *t-test* or two-way ANOVA and Tukey’s multiple comparison test

To further validate the role of SLC6A4 during HQD treatment, we applied AOM/DSS to WT mice and SLC6A4-deficient mice and treated them with HQD. Compared to those of WT mice, the body weight of SLC6A4-deficient mice was lower (Fig. 4A) and the DAI was greater (Fig. 4B). The length of colon tissues in SLC6A4-deficient mice was decreased, and the number of tumors was increased (Fig. 4C-D). A disturbed distribution of glands in the colon tissues of SLC6A4-deficient mice and accentuated infiltration of inflammatory cells were observed via HE staining (Fig. 4E). In addition, ELISA revealed increased concentrations of serotonin (5-HT) and inflammatory factors in the colon tissues of SLC6A4-deficient mice compared to those in the colon tissues of WT mice (Fig. 4F). It was found through western blot assays that increased phosphorylation of NFκB p65 in SLC6A4-deficient mice resulted in enhanced expression of NLRP3 inflammasome-associated proteins (NLRP3, ASC, and Caspase1), which led to Cleaved-Caspase1 P20 and Cleaved-GSDMD (Fig. 4G).

### SLC6A4 downregulation represses the inhibitory effect of DS pretreatment on MODE-K cell pyroptosis-related proteins

**Fig. 5:**
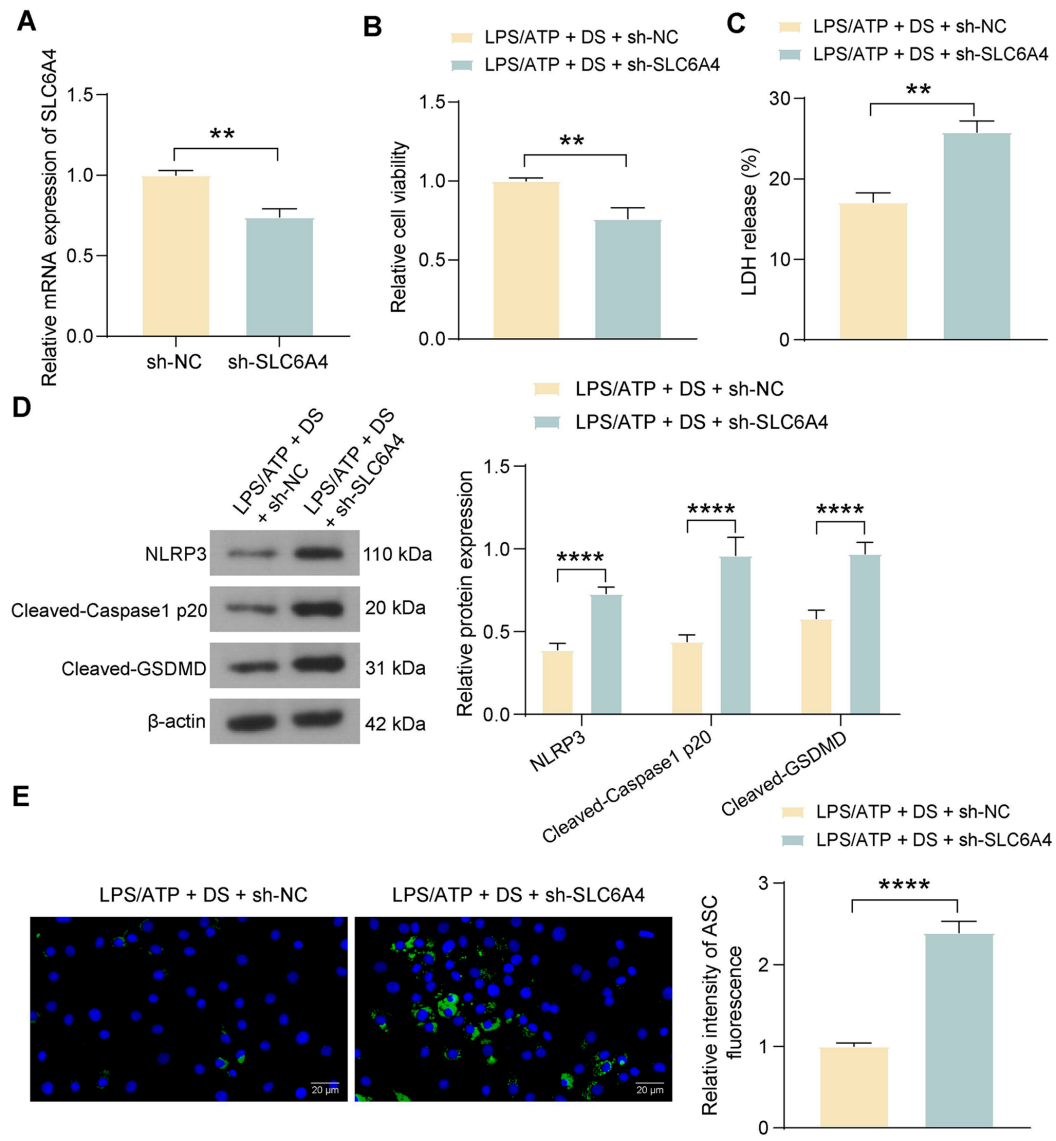
SLC6A4 downregulation promotes MODE-K cell pyroptosis treated with DS. MODE-K cells were infected with sh-SLC6A4 or sh-NC, pre-treated with DS, and induced with LPS-ATP. (A) Detection of SLC6A4 mRNA expression in MODE-K cells after infection by RT-qPCR. (B) MODE-K cell viability was examined using a CCK-8 assay. (C) Measurement of LDH release. (D) The expression of NLRP3, Cleaved-Caspase1 P20, and Cleaved-GSDMD in MODE-K cells was examined using a western blot. (E) Immunofluorescence detection of ASC expression in MODE-K cells. The data are presented as the means ± SD (*n* = 3). **p < 0.01, ****p < 0.0001. The data were analyzed by unpaired *t-test* or two-way ANOVA and Tukey’s multiple comparison test

To validate the effect of SLC6A4 on MODE-K cell pyroptosis in vitro, we infected MODE-K cells with lentivirus harboring sh-SLC6A4 or sh-NC and validated them by RT-qPCR (Fig. 5A). The effect of SLC6A4 downregulation on the pyroptosis of DS-pretreated MODE-K cells was subsequently examined. A CCK-8 assay showed that the viability of MODE-K cells was reduced after SLC6A4 downregulation following LPS/ATP induction (Fig. 5B). Depletion of SLC6A4 in MODE-K cells pretreated with DS and induced by LPS/ATP resulted in increased LDH release (Fig. 5C). Western blot assays revealed increased expression of the NLRP3, Cleaved-Caspase1 P20, and Cleaved-GSDMD proteins in MODE-K cells in response to SLC6A4 loss (Fig. 5D). In addition, the results of the immunofluorescence assay also showed that SLC6A4 downregulation exacerbated the pyroptosis in MODE-K cells (Fig. 5E).

## Discussion

As the third most common cancer and the second most deadly malignancy, colorectal cancer was estimated to cause 1.9 million incidences and 0.9 million deaths worldwide in 2020 (Xi and Xu 2021). HQD, a classic prescription for diarrhea in Chinese medicine treatment, has been revealed to ameliorate irinotecan-induced gastrointestinal toxicity and enhance its anticancer therapeutic efficacy (Wang et al. 2017; Xu et al. 2021). In this study, we demonstrated that HQD could be used to treat AOM/DSS-induced CAC through the regulation of SLC6A4. SLC6A4 reduced the levels of serotonin, thus blocking the NFκB-mediated NLRP3/Caspase1/GSDMD pathway.

HQD has been revealed to be capable of treating multiple disorders, including diabetic liver injury (Xu et al. 2022), nonalcoholic fatty liver disease (Yan et al. 2023), and Parkinson’s disease (Gao et al. 2022). Furthermore, the administration of HQD significantly inhibited the severity of 2,4,6-trinitrobenzenesulfonic acid-induced colitis in a dose-dependent manner (30–120 mg/kg) (Zou et al. 2015). In this study, we used AOM/DSS to induce CAC in mice and evaluated the therapeutic effects of HQD on a CAC animal model, with mesalazine serving as a positive control. HQD has been recently found to suppress colonic inflammation, attenuate DSS-induced clinical manifestations, reverse colon length shortening, and reduce histological injury in mice (Li et al. 2022; Mo et al. 2022). Here, we further observed that similar to mesalazine, HQD exhibited an antitumor effect on mice induced with CAC. Although HQD has been shown to effectively inhibit DSS-induced colitis alone or by synergizing with *Radix Actinidiae chinensis*, this main function was associated with altering the composition of the intestinal flora and modifying the proportion of T-cell subsets in colorectal lymphoid tissues (Huang et al. 2022a, b). Therefore, the immunoregulatory function of HQD needs to be further explored and verified.

To determine the biomolecules involved, we combined two bioinformatics tools. It was thus found that SLC6A4 is a possible target of HQD. The released serotonin participates in peristalsis, secretion, and vasodilation in the gut, and the extracellular serotonin is subsequently transported into surrounding epithelial cells through SLC6A4 (Tatsuoka et al. 2022). Dysfunctional mucosal serotonin signaling has been implicated in heightened visceral sensitivity and altered motility in patients with inflammatory bowel disease and animal models (Bertrand et al. 2010). It has been reported that compared with the control group, DSS treatment downregulated the expression of SLC6A4 in the colon (Wang et al. 2020). Our in vivo observation revealed consistent results in which AOM-DSS reduced the expression of SLC6A4 and enhanced the levels of serotonin in the colon of mice. Serotonin induced an inflammatory response and cell apoptosis in corneal epithelial cells by activating NFκB signaling (Zhang et al. 2019). Furthermore, because NFκB signaling was closely related to CAC (Tajasuwan et al. 2022) and can be regulated by HQD (Chen et al. 2018; Li et al. 2019), we investigated the downstream effectors of serotonin-mediated NFκB signaling.

It is well known that LPS-induced activation of the TLR4/NF-κB pathway is a priming step for the activation of the NLRP3 inflammasome (Perera et al. 2018; Wagatsuma and Nakase 2020). After that, inflammatory Caspase1 was activated in response to microbial infection and danger signals, thereby cleaving GSDMD to generate an N-terminal cleavage product (GSDMD-N) that triggers pyroptosis and the release of inflammatory cytokines, such as IL-1β (Liu et al. 2016). Recently, pyroptosis has been implicated in CAC (Gong et al. 2022). Therefore, we examined the protein expression of p-NFκB p65, NLRP3, Cleaved-Caspase1 P20, Cleaved-GSDMD, and ASC and the levels of IL-1β and IL-18 in the colon tissues of mice. AOM/DSS induced the NFκB signaling and the NLRP3/Caspase1/GSDMD pathway, which were blocked by the HQD treatment. Similar results were also obtained in MODE-K cells induced with LPS/ATP. Likewise, neferine inhibited LPS-ATP-induced endothelial cell pyroptosis by blocking the NLRP3/Caspase1 signaling pathway (Tang et al. 2019). More relevantly, NEK7 interacted with NLRP3 to activate the NLRP3 inflammasome activation, thereby enhancing the pyroptosis in MODE-K cells and DSS-induced chronic colitis in mice (Chen et al. 2019). Furthermore, we substantiated that the depletion of SLC6A4 mitigated the antitumor and anti-inflammatory effects of HQD in vivo. In addition, cytotoxicity and pyroptosis were enhanced in MODE-K cells infected with sh-SLC6A4 and treated with DS. However, due to the complex composition and numerous targets of herbs, we need to continue to explore the bioactive compounds in depth in subsequent experimental studies.

## Conclusion

**Fig. 6:**
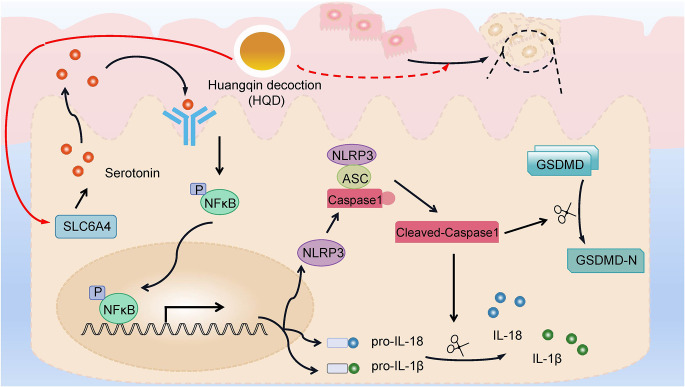
A schematic drawing of HQD in CAC. Activation of SLC6A4 expression by HQD decreased the secretion of serotonin. After serotonin secretion was reduced, the NFκB pathway-induced NLRP3/Caspase1/GSDMD pyroptosis pathway was blocked, which alleviated CAC.

HQD suppressed CAC in mice by decreasing cell pyroptosis and the release of inflammatory factors through the SLC6A4-mediated NLRP3/Caspase1/GSDMD signaling pathway (Fig. 6). In brief, our findings provide a theoretical basis for the treatment of CAC using HQD.

### Electronic supplementary material

Below is the link to the electronic supplementary material.

Supplementary Material 1

## Data Availability

No datasets were generated or analysed during the current study.
