# Correction to: Huang Qin decoction increases SLC6A4 expression and blocks the NFκB-mediated NLRP3/Caspase1/GSDMD pathway to disrupt colitisassociated carcinogenesis

**DOI:** 10.1007/s10142-024-01343-w

**Published:** 2024-04-25

**Authors:** Yili Tao, Lai Wang, Xiaofeng Ye, Xin Qian, Danye Pan, Xiaoyu Dong, Qian Jiang, Po Hu

**Affiliations:** 1 Department of Gastroenterology, Changzhou Hospital of Traditional Chinese Medicine, Changzhou, Jiangsu 213000 P.R. China; 2 Digestive Disease Diagnosis and Treatment Center of Integrated Traditional Chinese and Western Medicine, Changzhou Hospital of Traditional Chinese Medicine, Changzhou, Jiangsu 213000 P.R. China; 3 Department of Pulmonary Diseases, Changzhou Hospital of Traditional Chinese Medicine, Changzhou, Jiangsu 213000 P.R. China

**Correction to: Functional & Integrative Genomics (2024)**

10.1007/s10142-024-01334-x

The article Huang Qin decoction increases SLC6A4 expression and blocks the NFκB-mediated NLRP3/Caspase1/GSDMD pathway to disrupt colitis-associated carcinogenesis, written by Yili Tao, Lai Wang, Xiaofeng Ye, Xin Qian, Danye Pan, Xiaoyu Dong, Qian Jiang and Po Hu, was originally published online on the publisher’s internet portal on 12 March 2024 without open access. With the author(s)’ decision to opt for Open Choice the copyright of the article changed on 27 March 2024 to © The Author(s) 2024 and the article is forthwith distributed under a Creative Commons Attribution 4.0 International License, which permits use, sharing, adaptation, distribution and reproduction in any medium or format, as long as you give appropriate credit to the original author(s) and the source, provide a link to the Creative Commons licence, and indicate if changes were made. The images or other third party material in this article are included in the article’s Creative Commons licence, unless indicated otherwise in a credit line to the material. If material is not included in the article’s Creative Commons licence and your intended use is not permitted by statutory regulation or exceeds the permitted use, you will need to obtain permission directly from the copyright holder. To view a copy of this licence, visit http://creativecommons.org/licenses/by/4.0.

The original article has been corrected.

