# Supplementary material for: Huang Qin decoction increases SLC6A4 expression and blocks the NFκB-mediated NLRP3/Caspase1/GSDMD pathway to disrupt colitis-associated carcinogenesis
Source: Funct Integr Genomics. 2024 Mar 12;24(2):55. doi: 10.1007/s10142-024-01334-x (PMC10927794; doi:10.1007/s10142-024-01334-x)

Western blot bands

Fig 2D-first

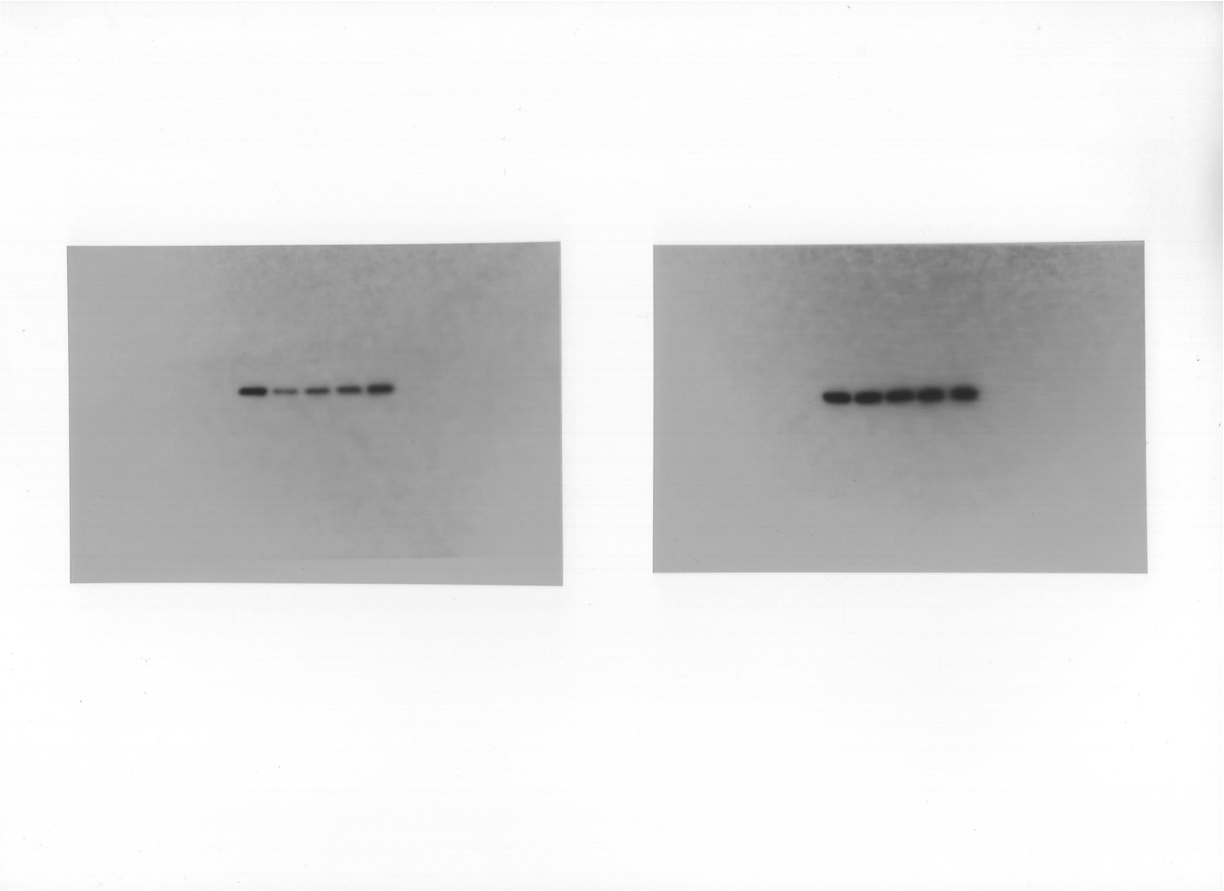

Fig 2D-second

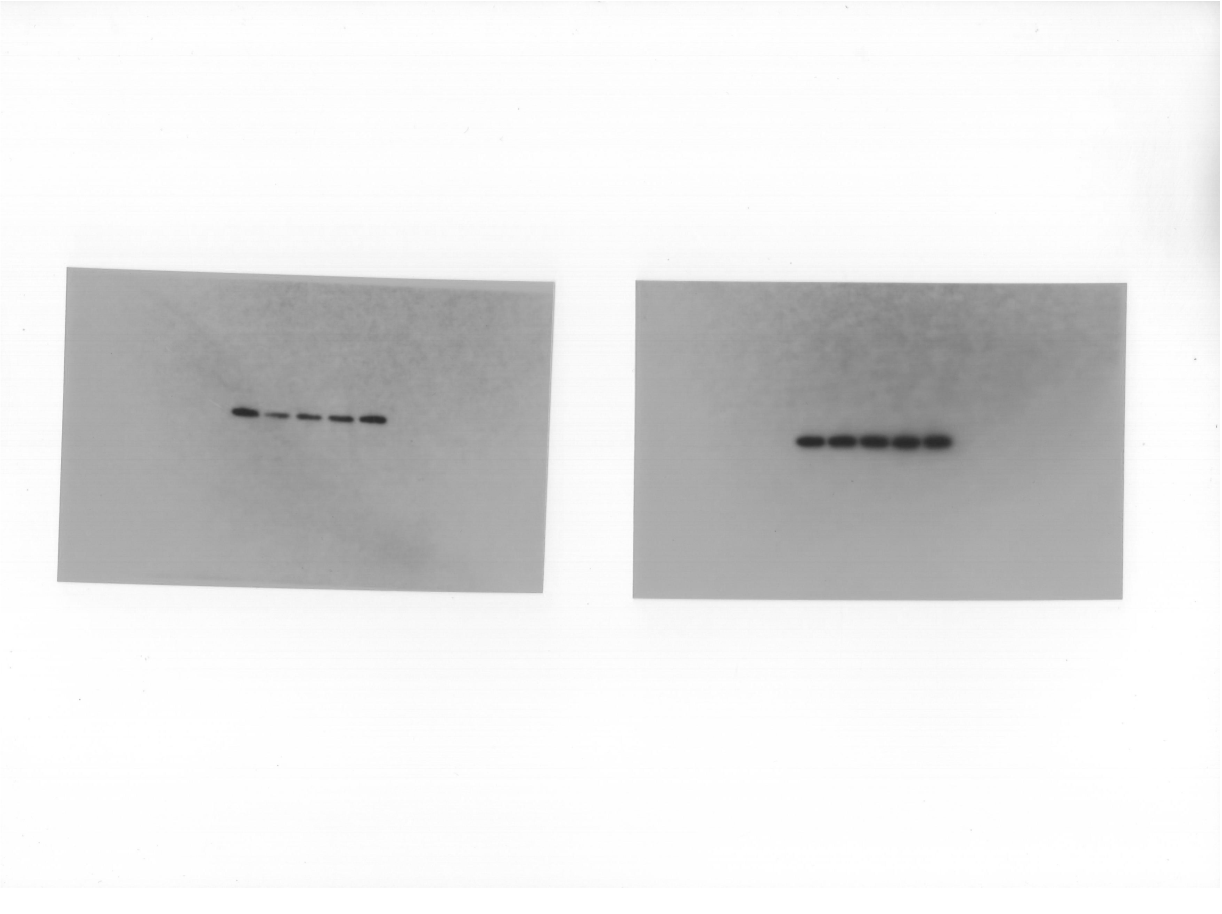

Fig 2D-third

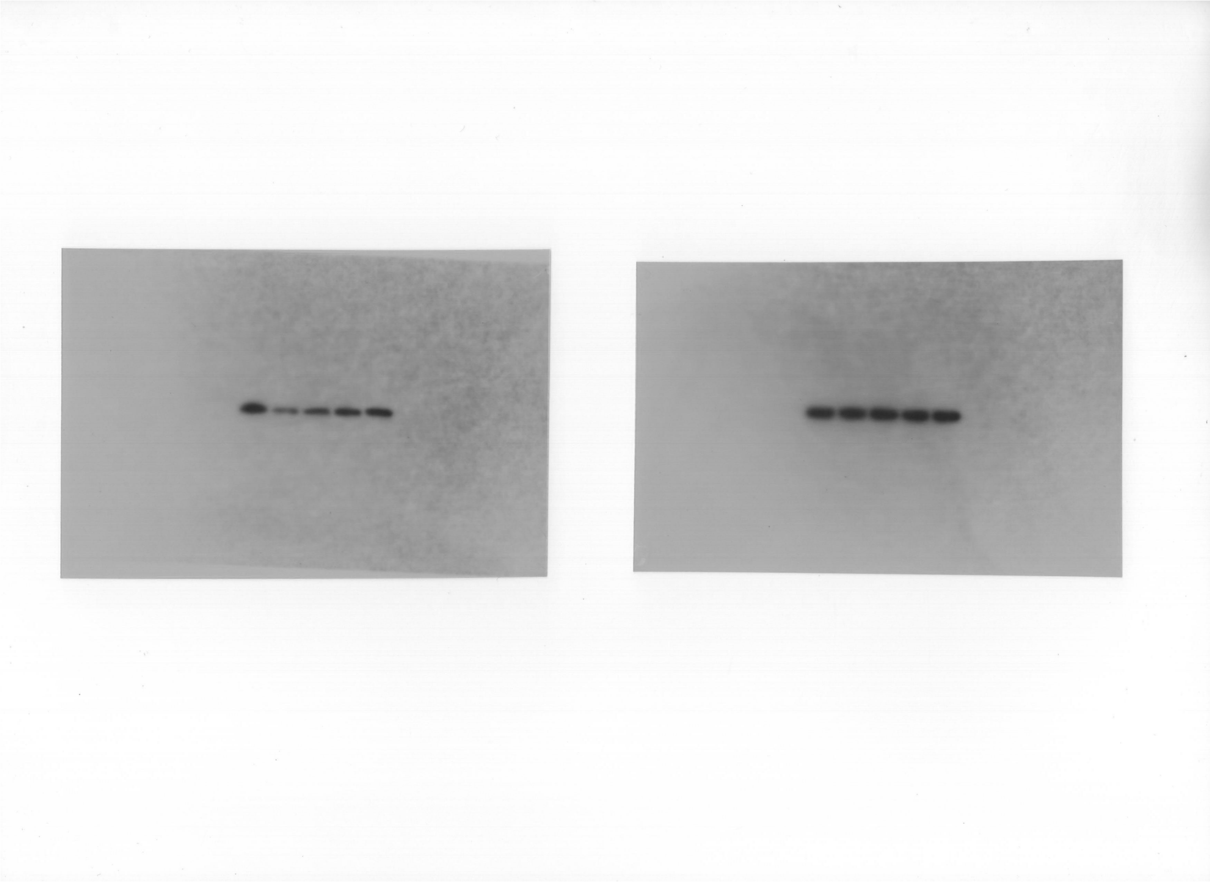

Fig 2D-fourth

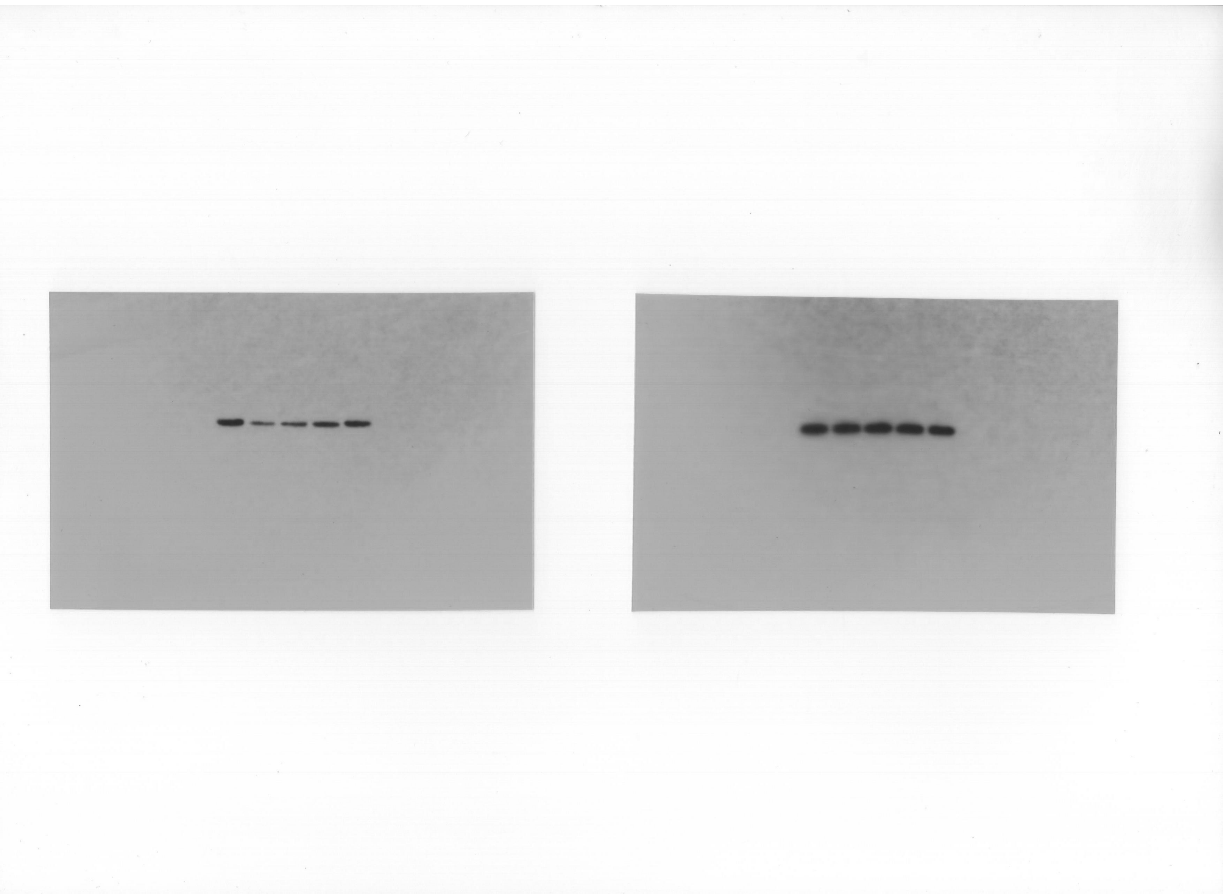

Fig 2D-fifth

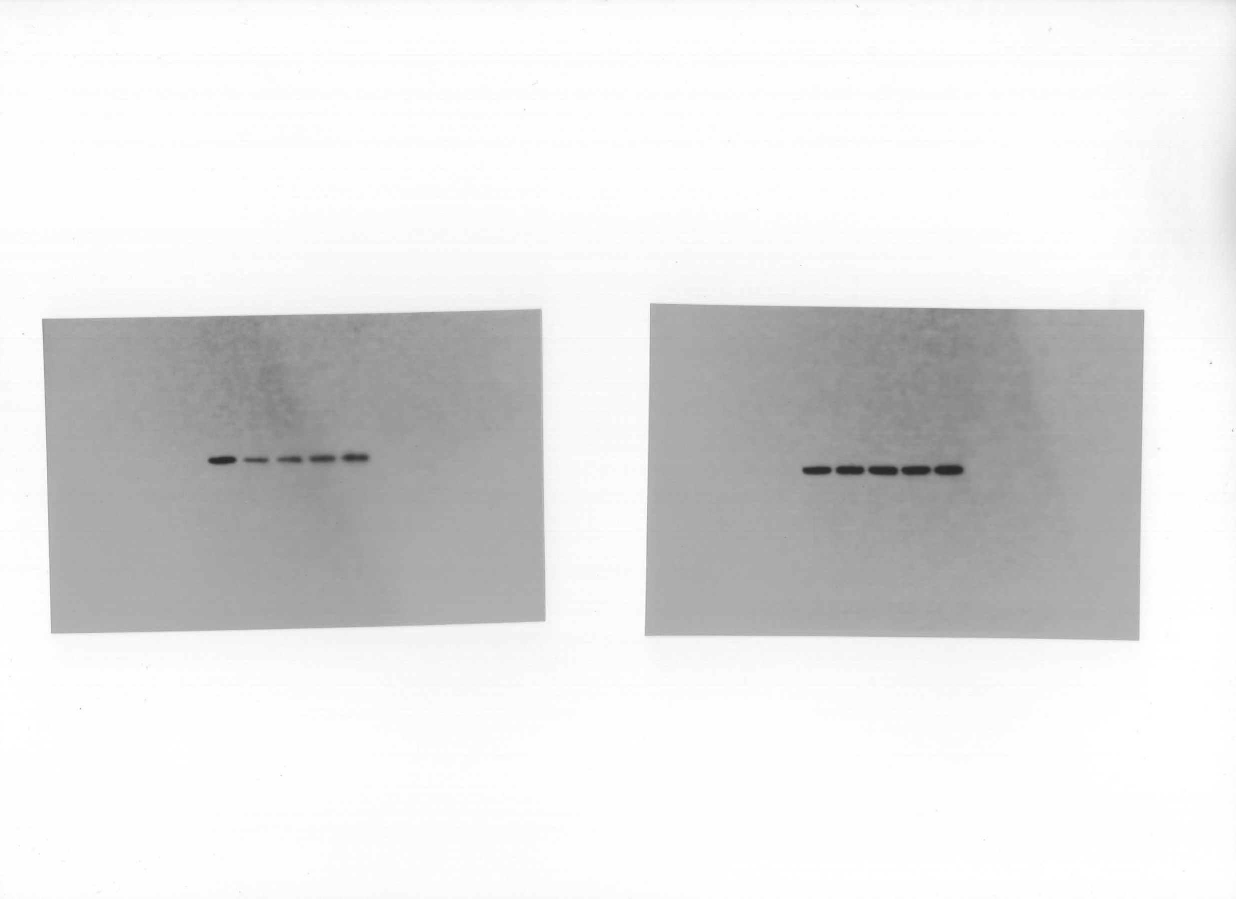

Fig 2D-sixth

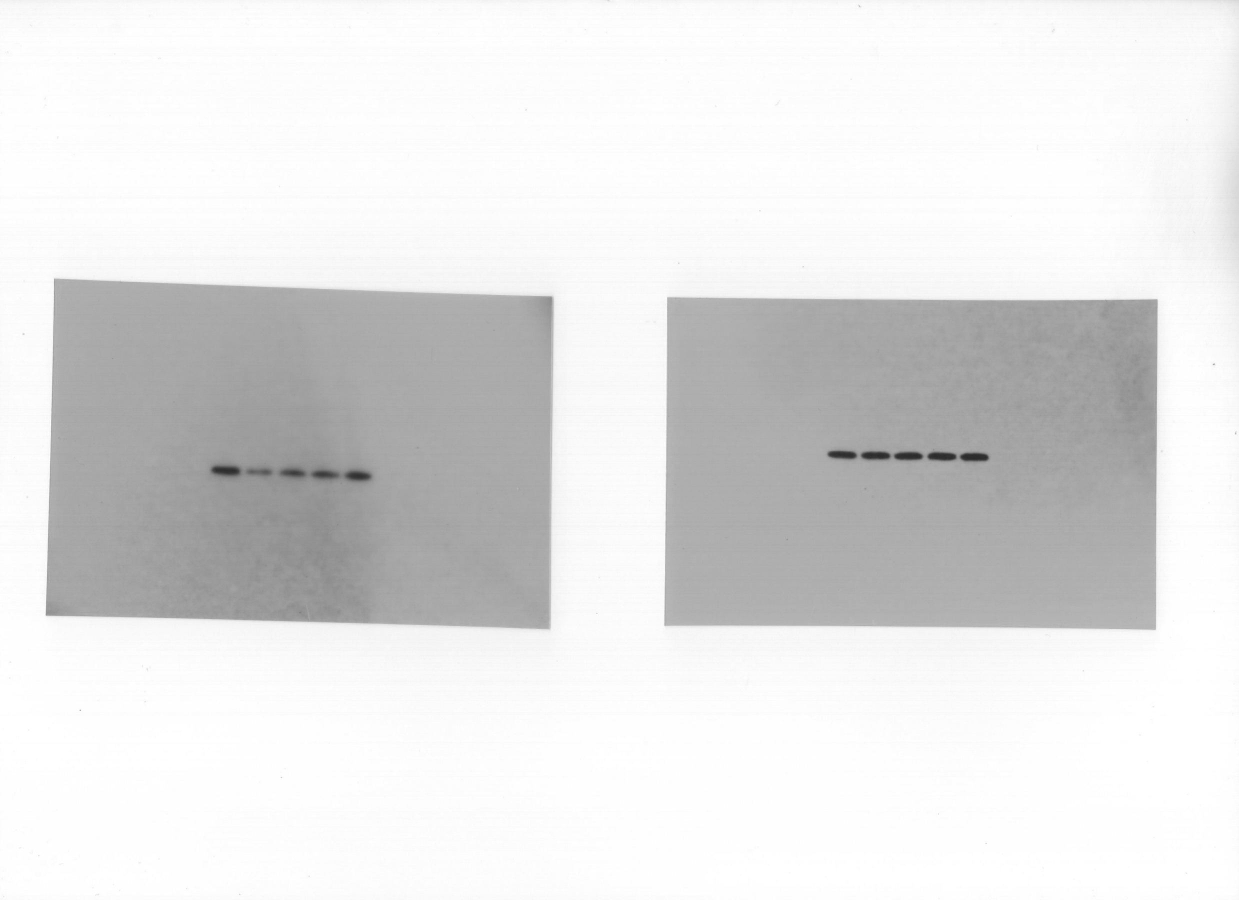

Fig 3A-first

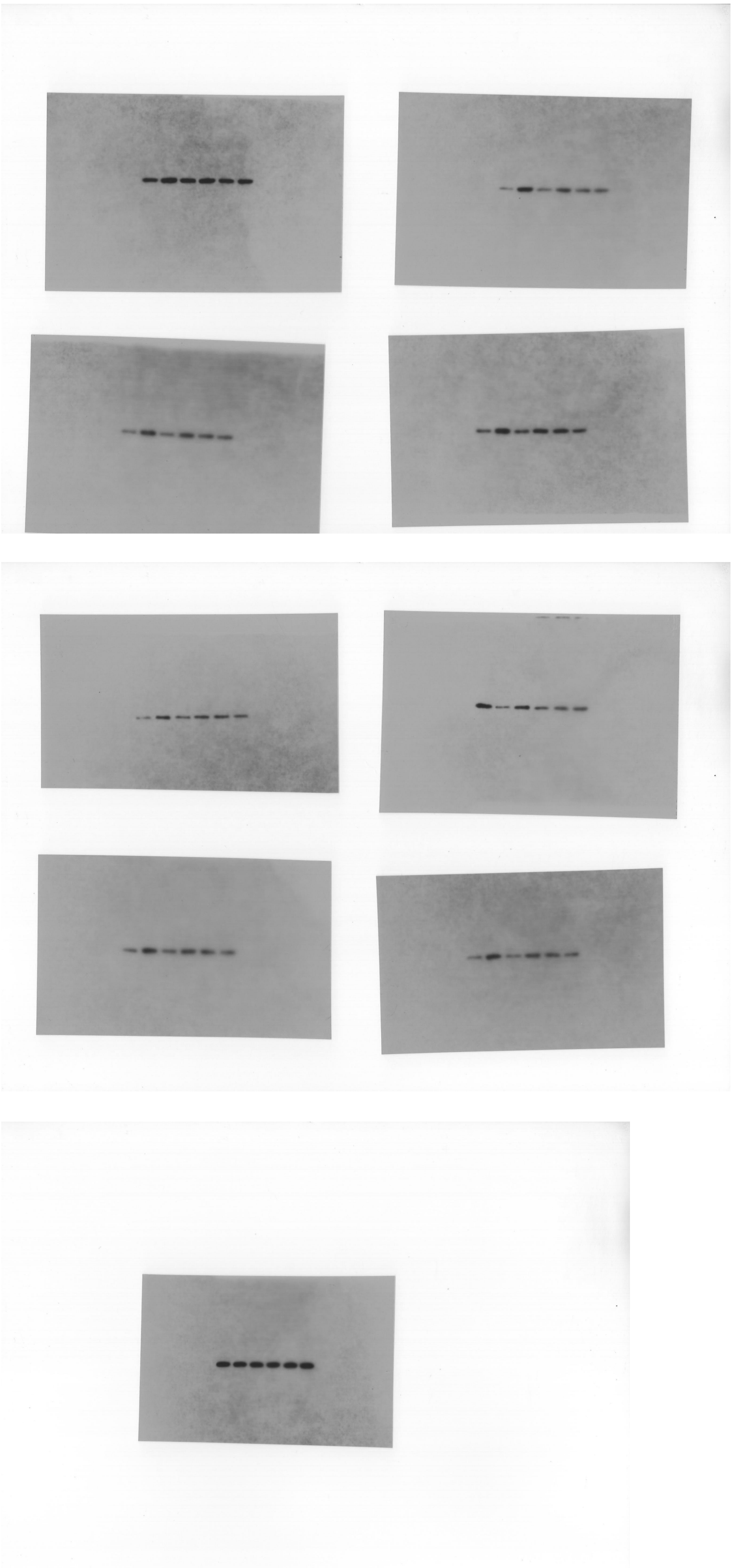

Fig 3A-second

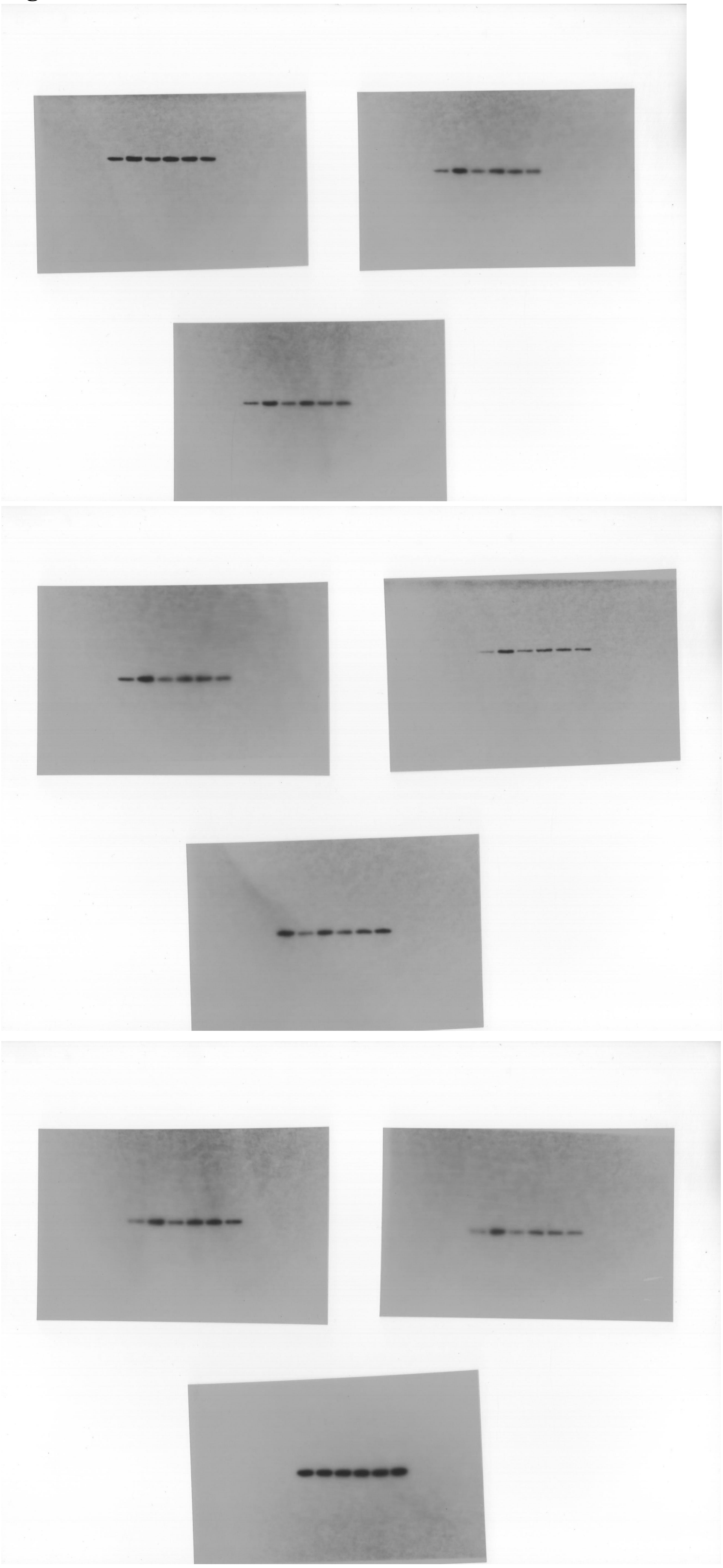

Fig 3A-third

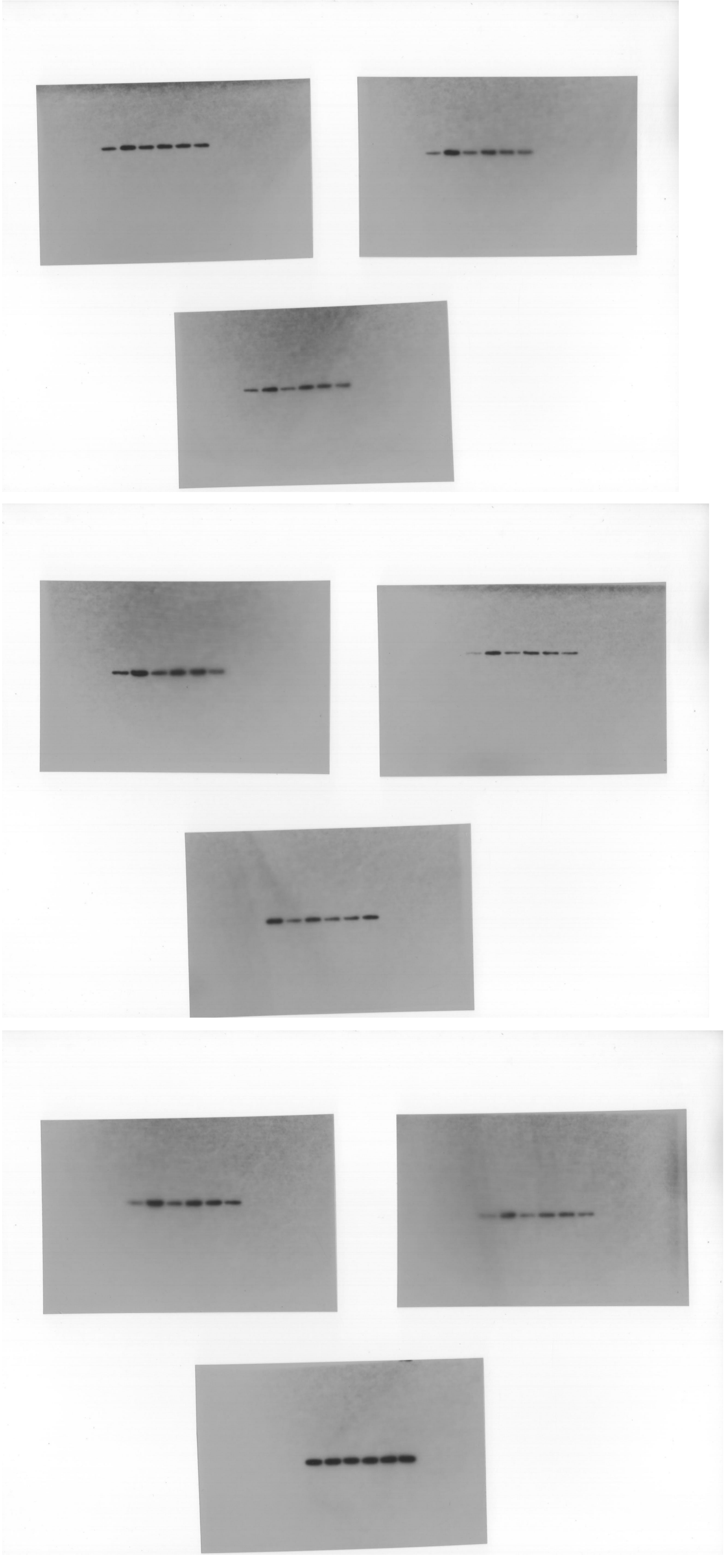

Fig 3A-fourth

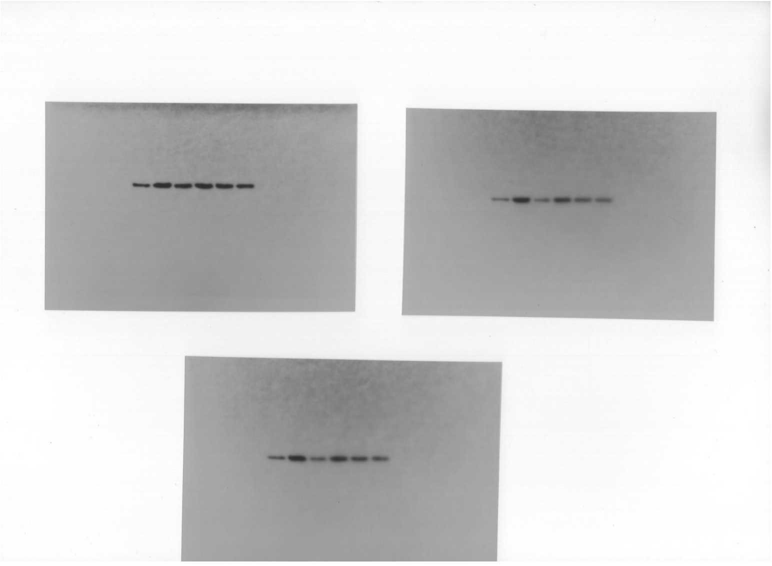

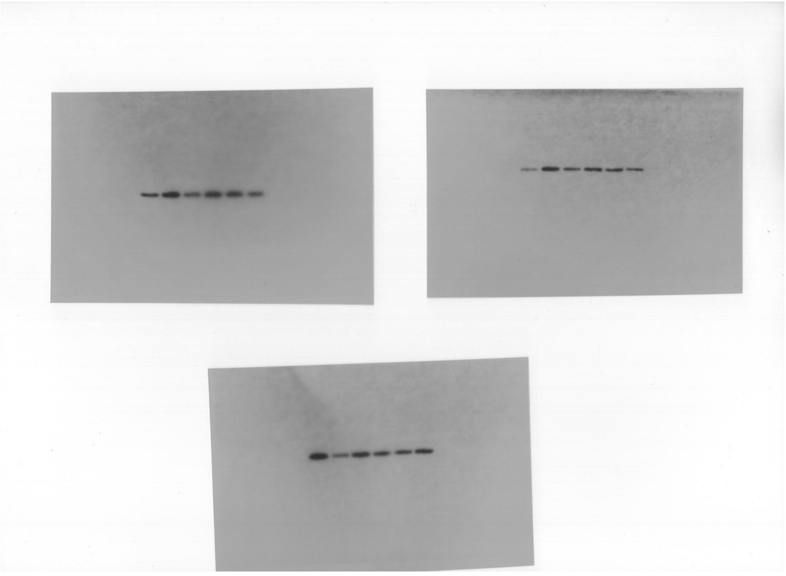

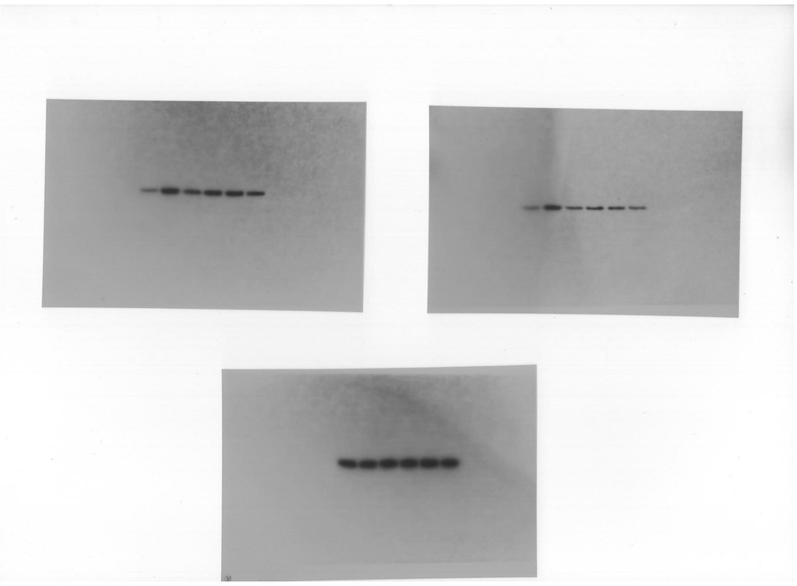

Fig 3A-fifth

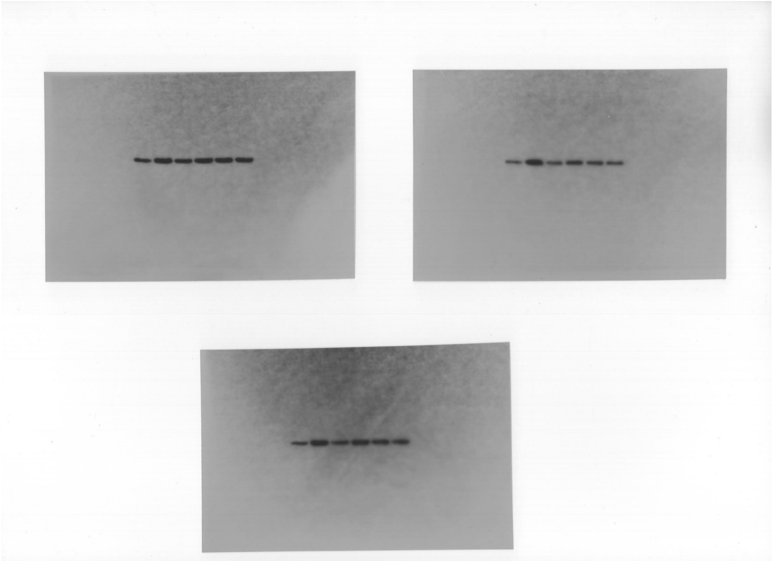

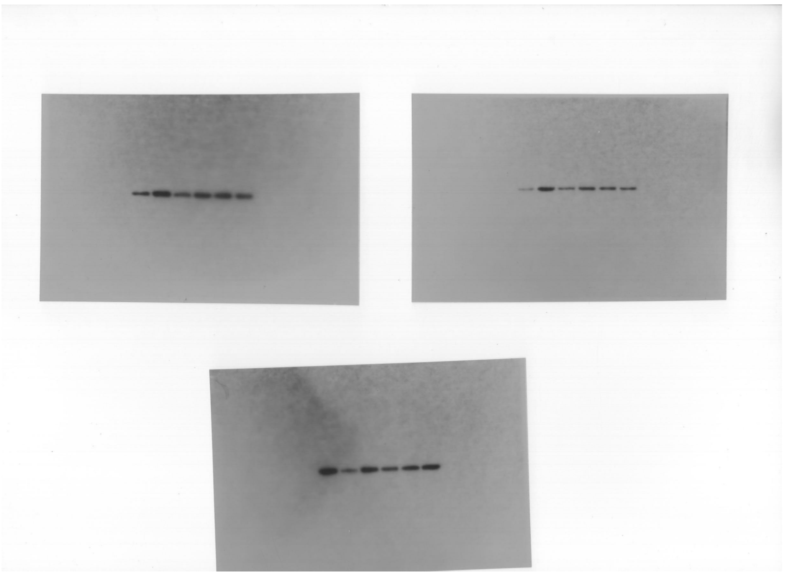

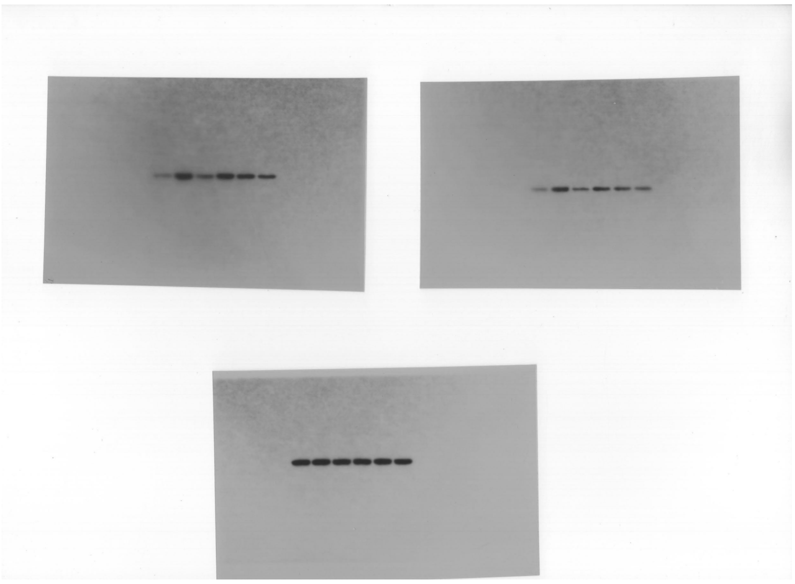

Fig 3A-sixth

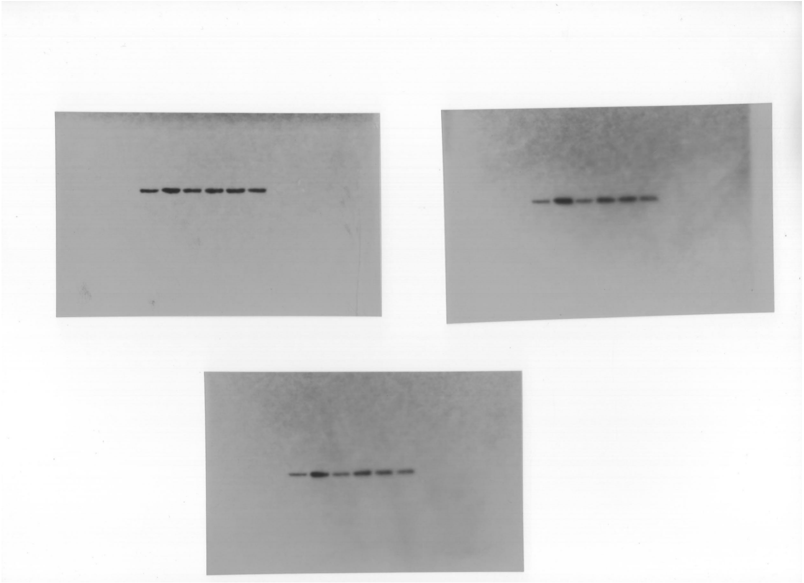

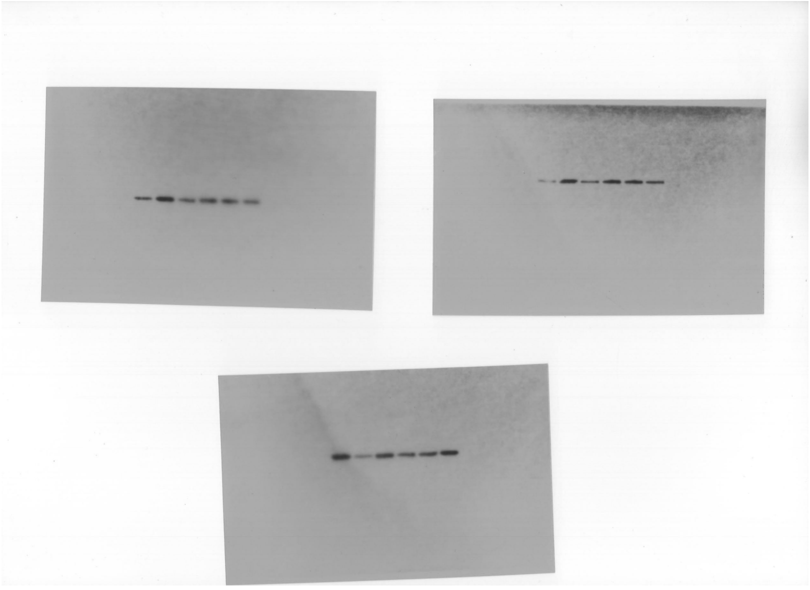

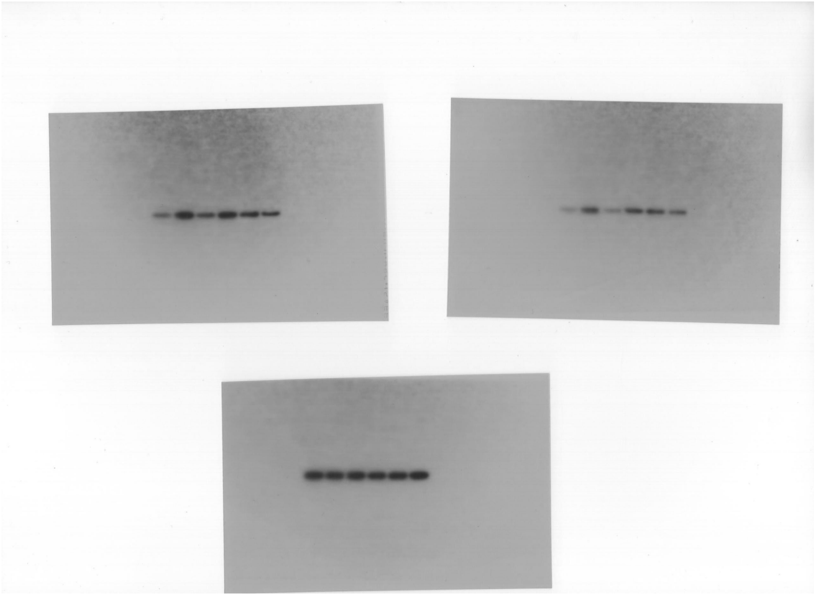

Fig 3E-first

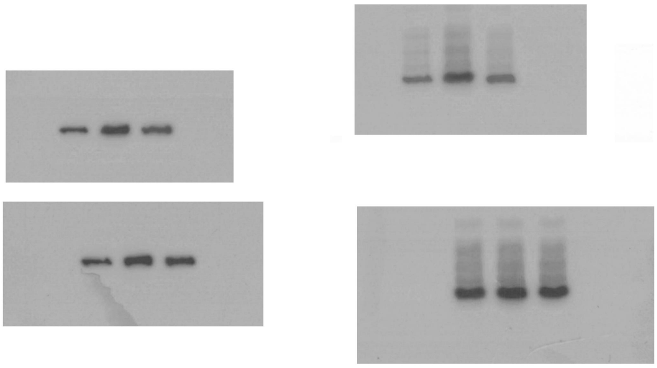

Fig 3E-second

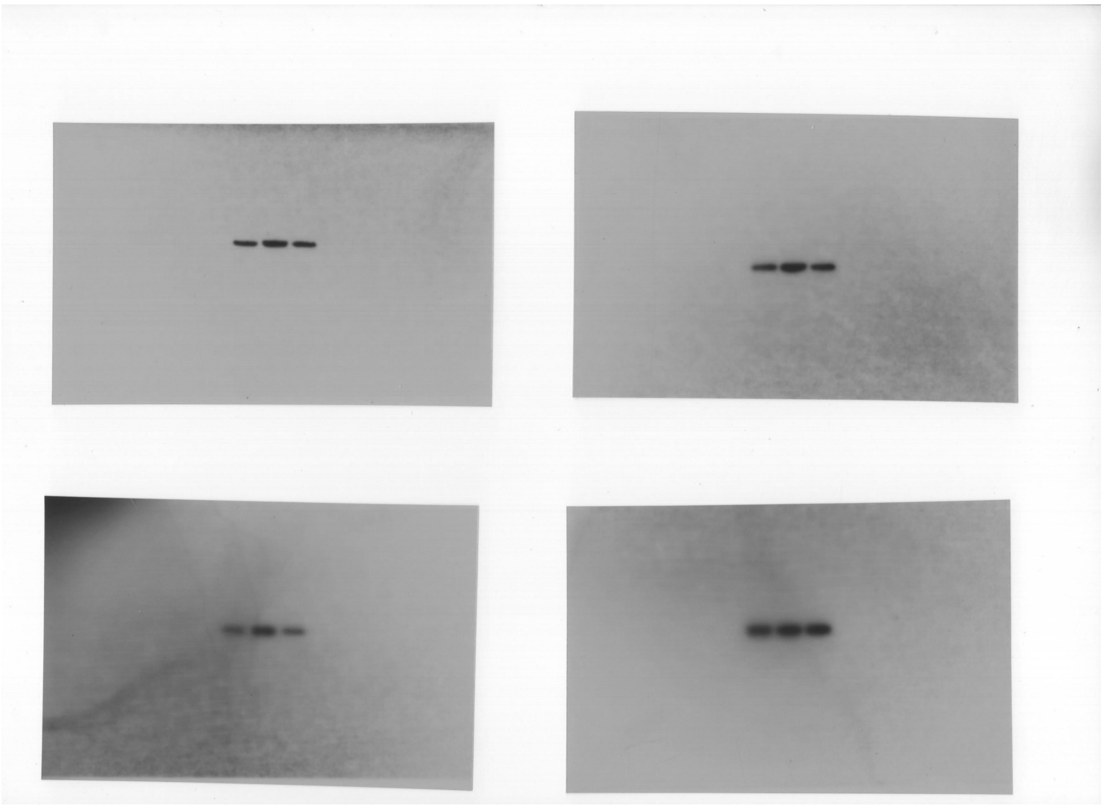

Fig 3E-third

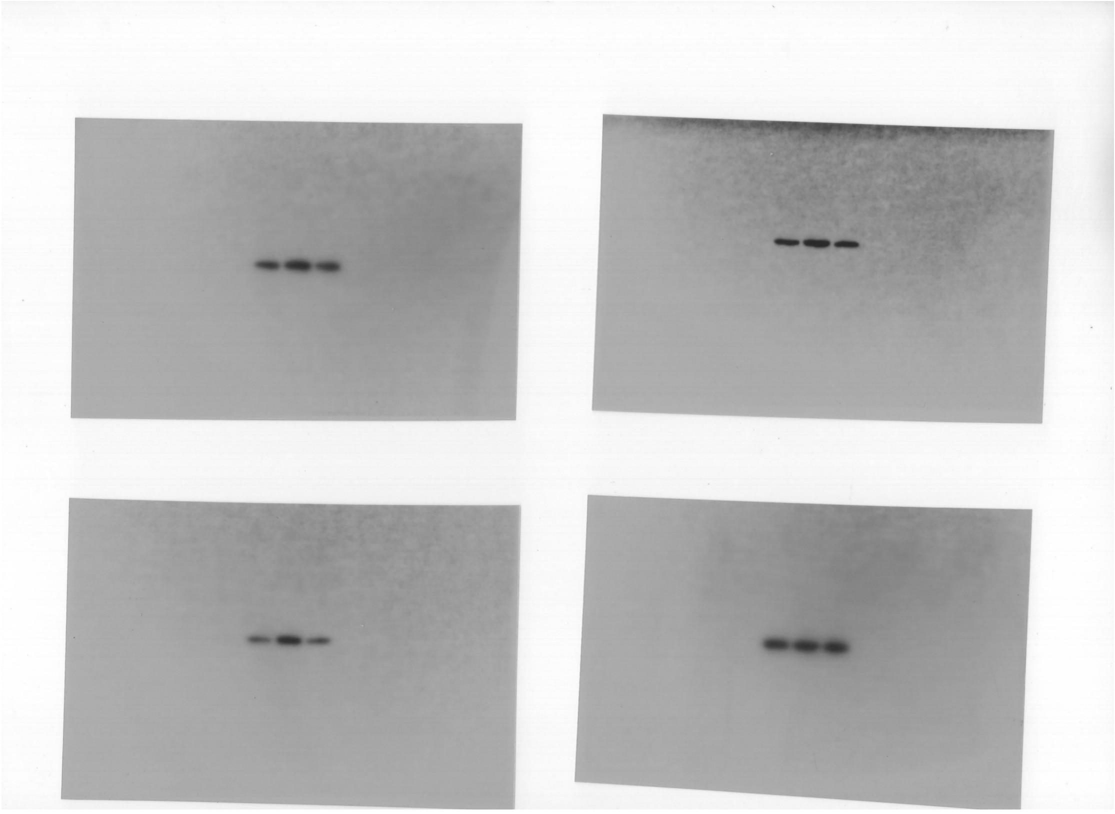

Fig 4G-first

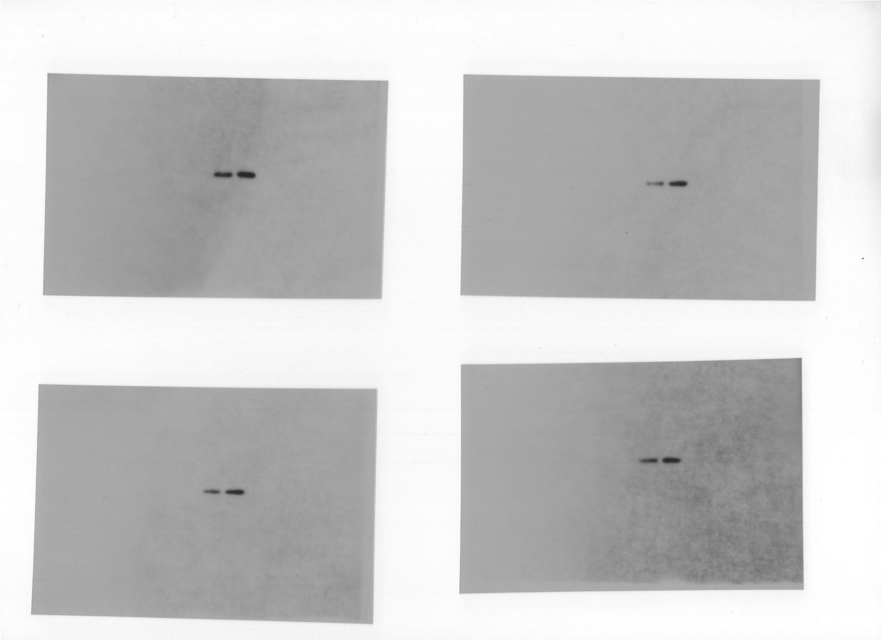

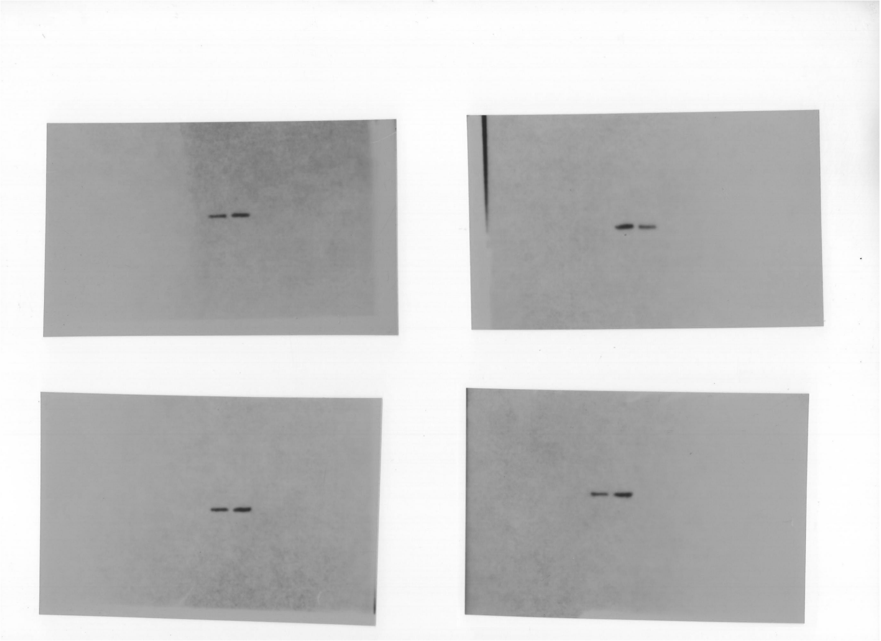

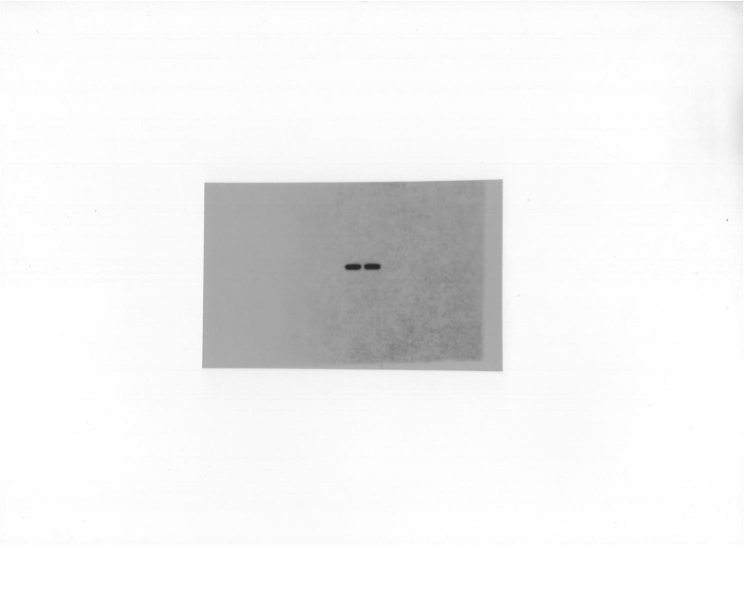

Fig 4G-second

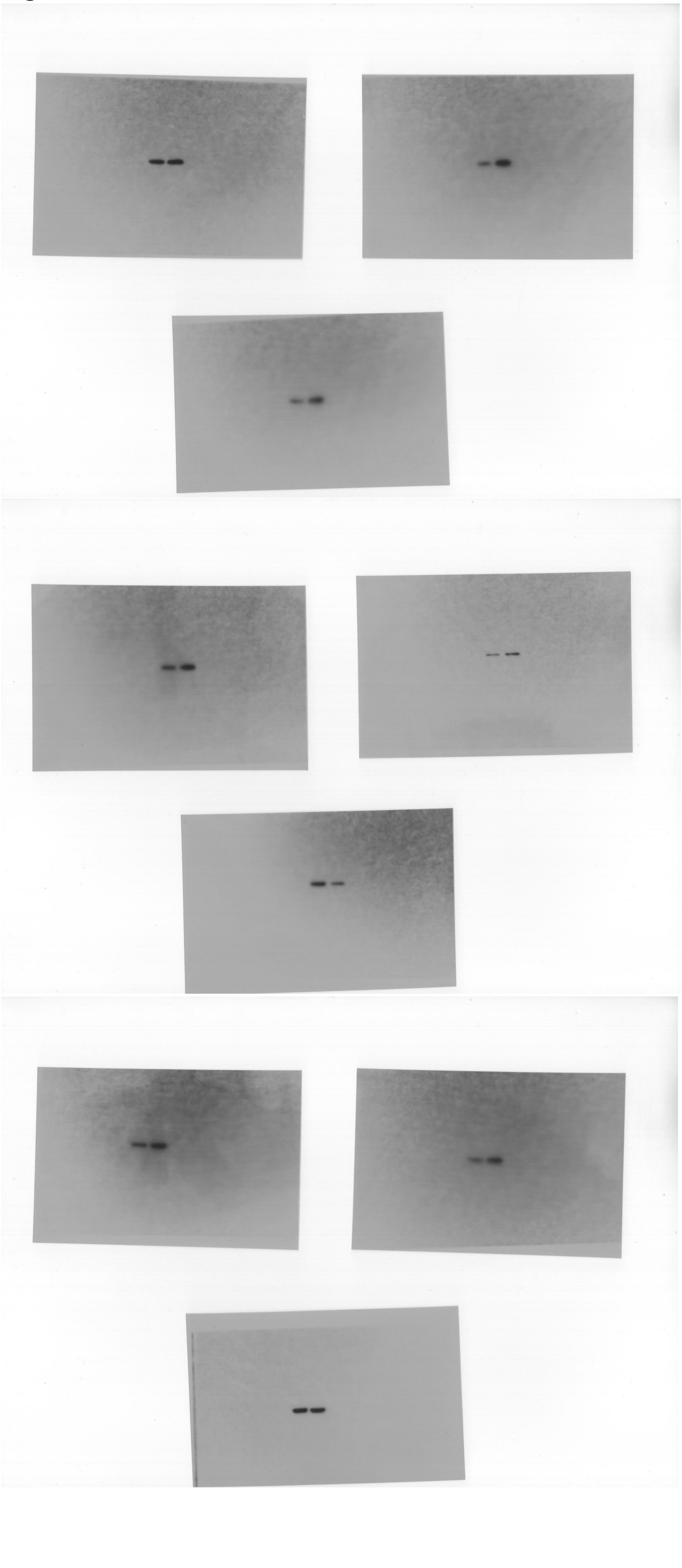

Fig 4G-third

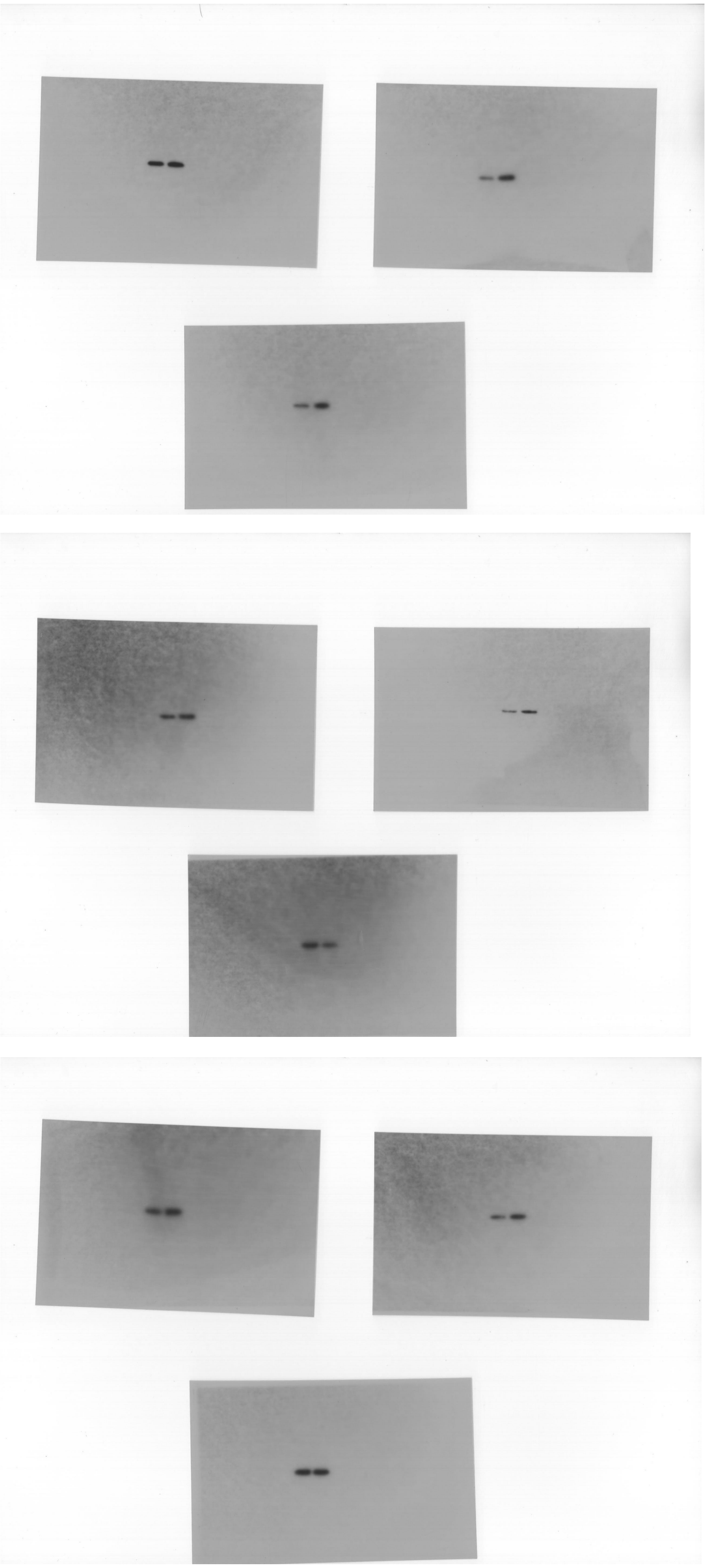

Fig 4G-fourth

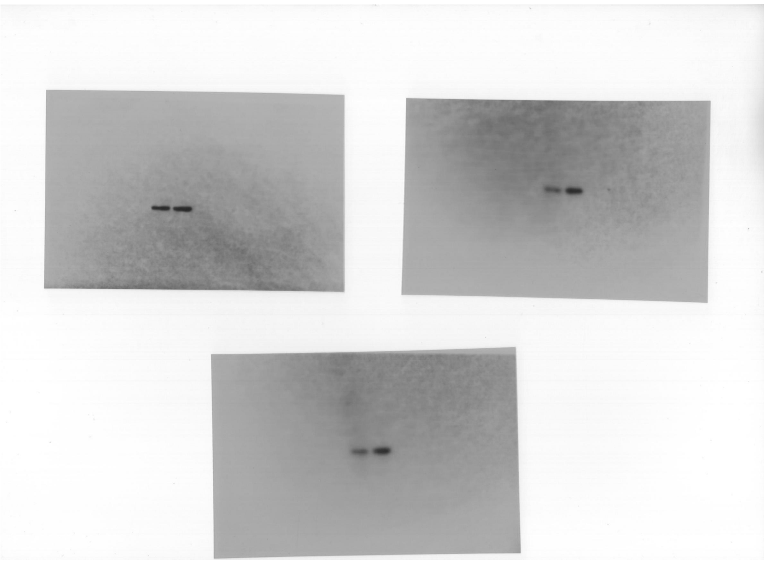

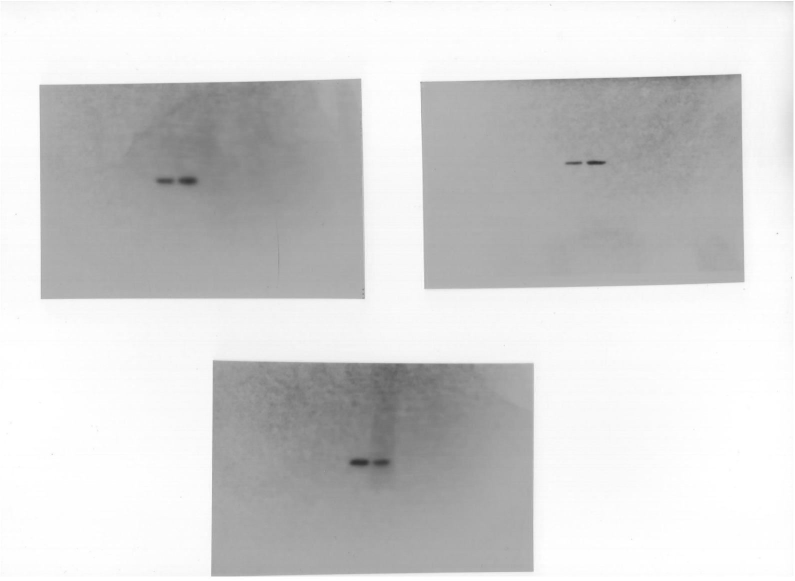

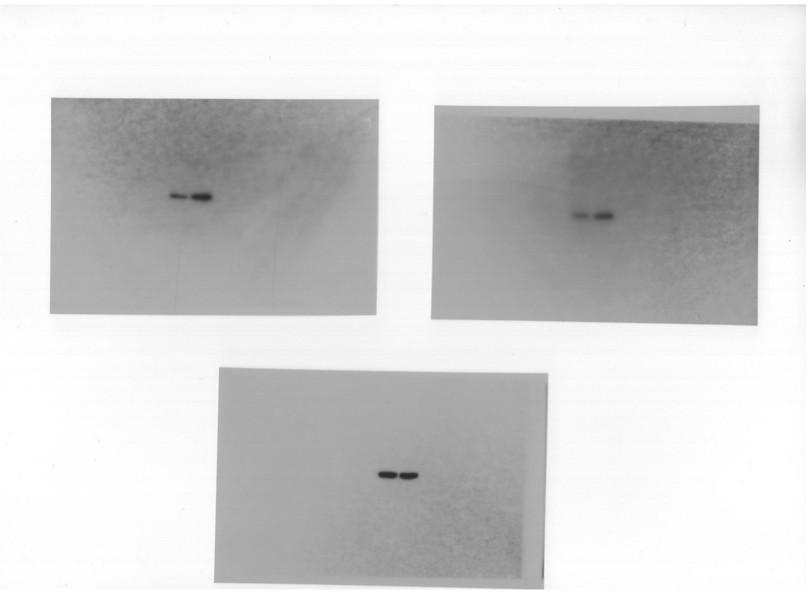

Fig 4G-fifth

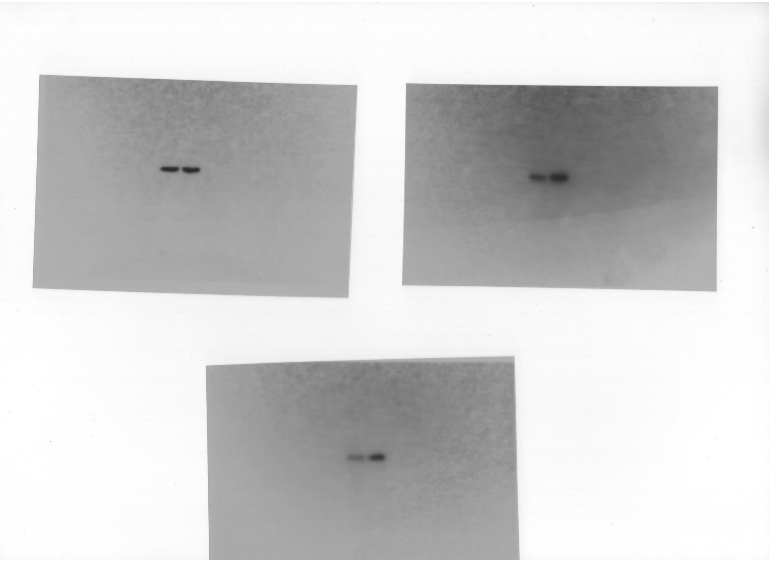

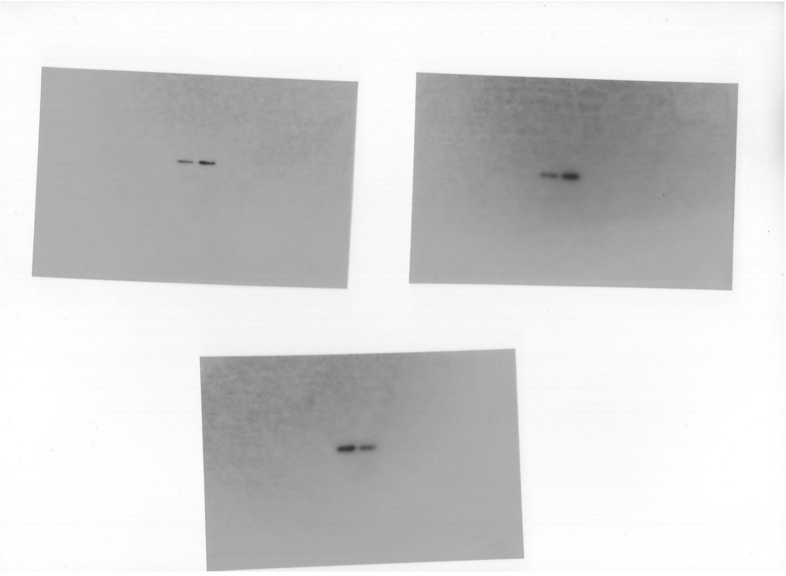

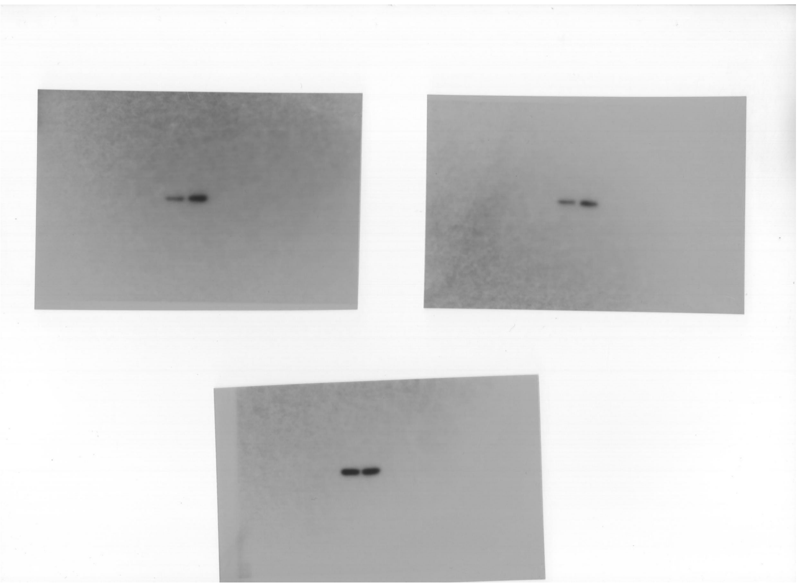

Fig 4G-sixth

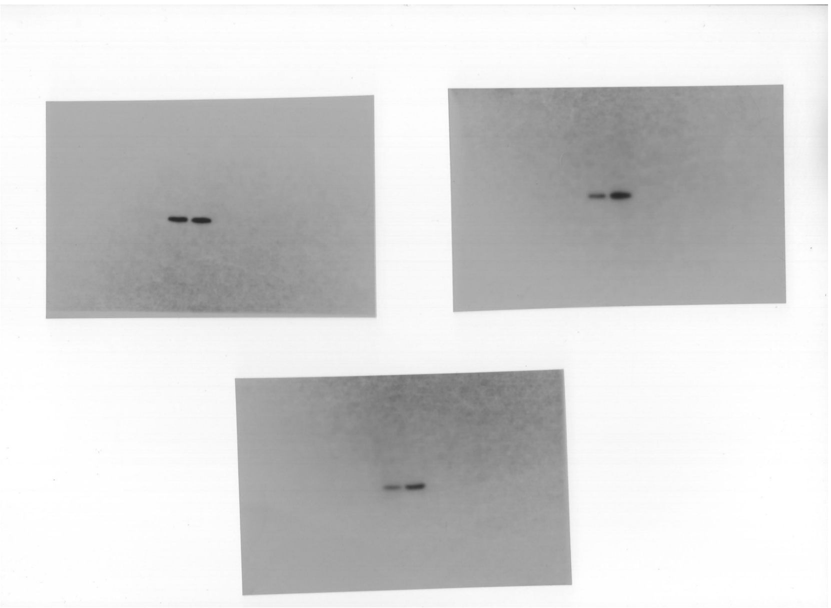

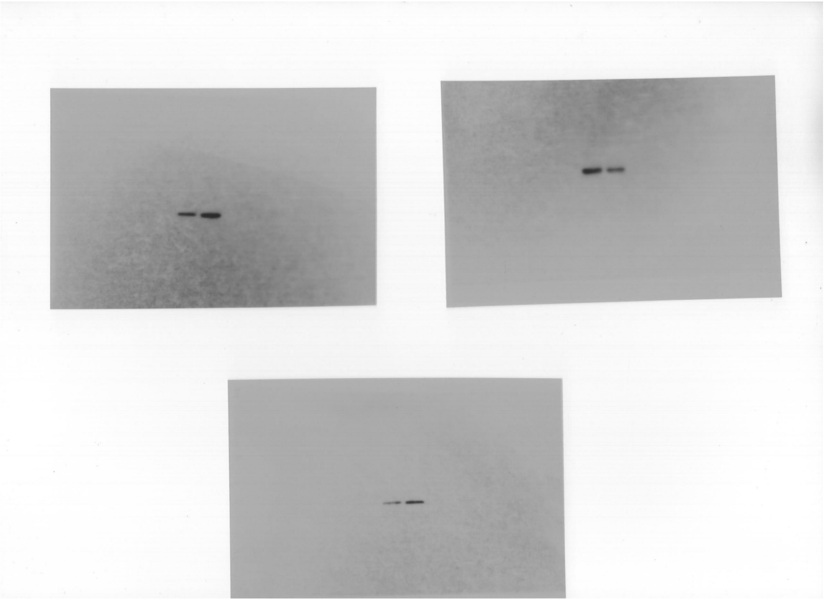

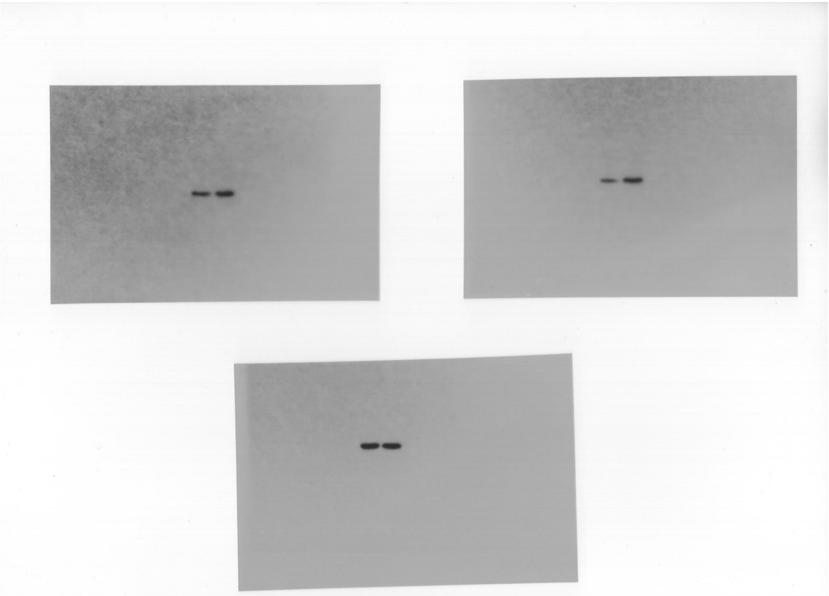

Fig 5D-first

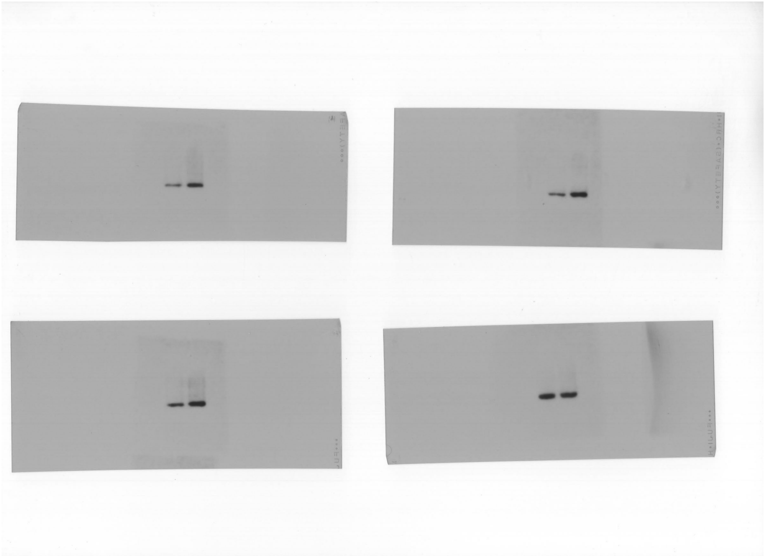

Fig 5D-second

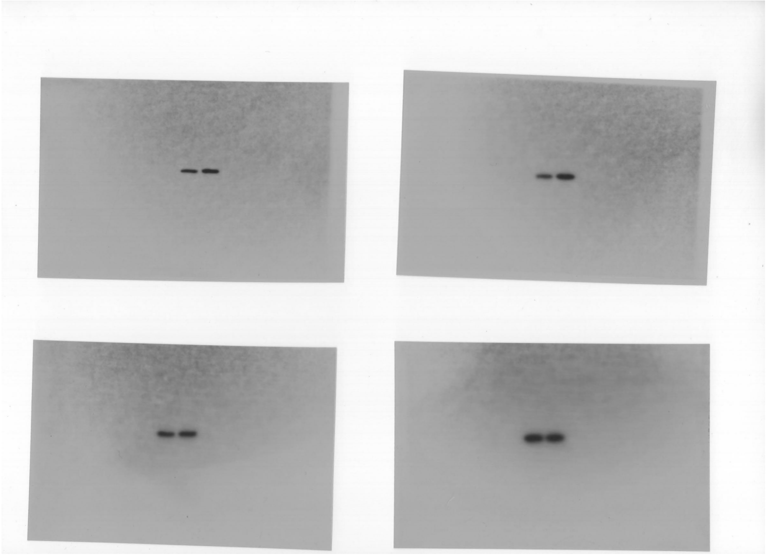

Fig 5D-third

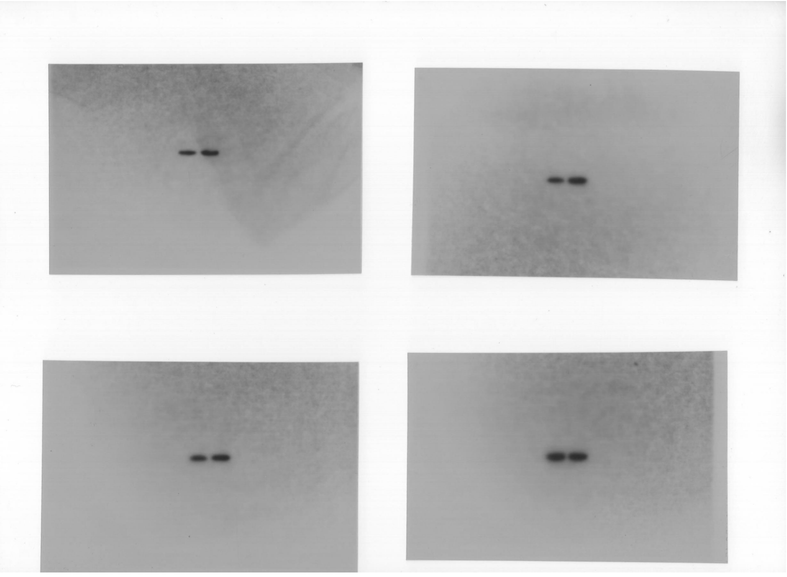

Supplement: Supplementary file 1 — Supplementary Material 1 [file 10142_2024_1334_MOESM1_ESM.pdf]
